# Sense-antisense gene-pairs in breast cancer and associated pathological pathways

**DOI:** 10.18632/oncotarget.6255

**Published:** 2015-10-28

**Authors:** Oleg V. Grinchuk, Efthymios Motakis, Surya Pavan Yenamandra, Ghim Siong Ow, Piroon Jenjaroenpun, Zhiqun Tang, Aliaksandr A. Yarmishyn, Anna V. Ivshina, Vladimir A. Kuznetsov

**Affiliations:** ^1^ Bioinformatics Institute, Agency for Science, Technology and Research (A*STAR), Singapore; ^2^ School of Computing Engineering, Nanyang Technological University, Singapore; ^3^ currently EM is working in RIKEN, Japan

**Keywords:** sense-antisense, breast cancer, prognostic, meta-analysis, GABPA

## Abstract

More than 30% of human protein-coding genes form hereditary complex genome architectures composed of sense-antisense (SA) gene pairs (SAGPs) transcribing their RNAs from both strands of a given locus. Such architectures represent important novel components of genome complexity contributing to gene expression deregulation in cancer cells. Therefore, the architectures might be involved in cancer pathways and, in turn, be used for novel drug targets discovery. However, the global roles of SAGPs in cancer pathways has not been studied. Here we investigated SAGPs associated with breast cancer (BC)-related pathways using systems biology, prognostic survival and experimental methods. Gene expression analysis identified 73 BC-relevant SAGPs that are highly correlated in BC. Survival modelling and metadata analysis of the 1161 BC patients allowed us to develop a novel patient prognostic grouping method selecting the 12 survival-significant SAGPs. The qRT-PCR-validated 12-SAGP prognostic signature reproducibly stratified BC patients into low- and high-risk prognostic subgroups. The 1381 SAGP-defined differentially expressed genes common across three studied cohorts were identified. The functional enrichment analysis of these genes revealed the GABPA gene network, including BC-relevant SAGPs, specific gene sets involved in cell cycle, spliceosomal and proteasomal pathways. The co-regulatory function of GABPA in BC cells was supported using siRNA knockdown studies. Thus, we demonstrated SAGPs as the synergistically functional genome architectures interconnected with cancer-related pathways and associated with BC patient clinical outcomes. Taken together, SAGPs represent an important component of genome complexity which can be used to identify novel aspects of coordinated pathological gene networks in cancers.

## INTRODUCTION

RNA transcripts of sense-antisense (SA) gene pairs (SAGPs) represent a large subset of the human transcriptome varying from 30 to 50 % at all loci [1-3]. The gene partners of an SAGP (i) are located on different strands of a chromosome, (ii) share a common locus and (iii) are transcribed in opposite directions. Therefore, SAGPs represent the natural genomic architectures evolutionarily organized in specific structural (and often functional) hereditary units. In terms of genetic architecture they can be classified into divergent (“head-to-head”), convergent (“tail-to-tail”) and embedded (“one inside another”) orientations [2, 4] comprising 29, 33 and 38%, respectively[5].

The physical interconnections of such paired genes indicate their evolutionary and functional relationships between them and specific control co-regulatory mechanisms [2, 4, 6]. One antisense transcript can lead either to activation or suppression of its sense transcript counterpart [2, 4]. Many reports documented the association of individual SAGPs with disease and cancer [7-9] (for a more detailed review refer to [10]), suggesting that SAGPs might be directly involved in disease [11, 12]. Global deregulated patterns of SA transcripts and gene pairs in cancers have also been well documented [5, 13-16]. In this context, a comprehensive approach to localize “hotspots” of deregulated antisense transcription [17], clarification of their global regulatory mechanisms and their involvement in pathobiological pathways in cancer could be *clinically* relevant.

Two approaches predominate the field of SAGP studies. The first approach is based on the detailed characterization of a single SAGP, focusing on diverse molecular mechanisms of SA transcription and post-transcription events and their involvement in cancer or other diseases. For example, a post-transcriptional mRNA stabilization mechanism has been found for p53 expression due to double-stranding p53 mRNA with the Wrap53 gene mRNA [8], which might be relevant in many cancers. High expression of the *MYCN* cis-antisense gene *NCYM* is associated with poor prognosis in neuroblastoma via promotion of production of anti-apoptotic protein Myc-nick [18]. Sharing a bidirectional promoter leads to coordination of gene expression levels for *BAL/BBAP* SAGP, providing optimal interaction of their protein products in chemoresistant, diffuse, large-cell lymphomas [19]. An advantage of this approach is that it can potentially provide alternative pharmaceutical strategies to activate/suppress the expression of well-known and important oncogenes/tumor suppressors. Specifically, disease-related individual SAGPs might represent a novel type of drug target for locus-specific, anti-sense modulation of abnormally activated genes of interest [20, 21]. The disadvantages of such “single SAGP” studies are: i) the lack of a complete physiological view at the level of global cellular regulation and ii) the unclear relative functional impact of the given SAGP in the context of the entire functioning set of SA gene pairs.

The second approach implies systematic study of SAGPs and their transcripts starting from the whole transcriptome scale with consequent specification of the specific subsets of transcripts/genes with common characteristics. This approach is aimed to unravel the general characteristics and mechanisms of SA phenomena (e.g., their common relative impact on the complexity of the transcriptome in disease and normal states, global association with transcription, posttranscriptional and posttranslational modifications) [5, 13, 16, 22, 23].

Here we studied the novel characteristics and possible coregulatory mechanisms of SAGPs in breast cancer (BC) using the second approach starting with transcriptome analysis.

BC is a highly heterogeneous disease with distinct morphological appearance and varied molecular features. The development and progression of a breast tumor is a complex and dynamic biological process. This complexity is determined by multiple genetic and molecular factors and components, including multiple genomic DNA aberrations (which can dramatically affect expression of large numbers of physically co-localized genes), global epigenetic changes and the regulatory effects of non-coding RNAs. Our understanding of tumorigenesis and related future therapeutic implications might substantially benefit from the integration of different components of genomic complexity and diverse omics data [24]. SAGPs and their products represent another component of genomic organization and molecular complexity and common molecular factors impacting BC tumorigenesis and tumors development [15, 16, 25]. The SAGPs are highly-populated complex architectures in the human genome and they may be patho-biologically important and clinically useful.

The main goal of this study is the consequent identification and characterization of the prognostically significant SAGPs in BCs, which importance in pathogenesis of cancers and in clinical oncology practice is currently under-estimated. We assumed that because SAGPs are evolutionary predetermined natural gene architectures, coordinated expression of their gene partners should be important for certain cellular functions and, therefore, might be involved in specific regulatory pathways in cells. In this context, studying SAGPs with deregulated expression profiles of their gene partners in specific pathologic BC subgroups/subtypes will help to clarify in which abnormally activated cancer pathways they could be predominantly involved. Here, we considered only SAGPs for which each gene partner encodes a protein (protein coding-coding SAGPs) because these SAGPs are much better annotated and more evolutionarily conserved than the other SAGP subclasses [5]. The expression patterns of both genes in an SAGP could be mutually or directionally co-regulated [21, 26], affecting the levels of both their RNA and protein products, which could significantly impact cell fate.

Additionally, we introduced a concept of the prognostic SAGPs-based signature as an important component of our entire meta-analysis workflow to identify and characterize SAGPs-associated deregulated molecular pathways and the potential regulatory factors of SAGPs in BC cells. We developed a computational approach for automatic identification of prognostic SAGPs using our original survival prediction model and feature selection algorithm. The algorithm implementation identified the refined SAGPs-associated BC patients survival subgroups, which in turn led to the discovery of the 1381 SAGP-defined differentially expressed genes (DEGs) and GABPA transcriptionally co-activated gene network comprising many BC-relevant SAGPs, as well as certain specific gene sets involved in the cell cycle, proteasome and spliceosome pathways. We demonstrated SAGPs as the synergistically functional genome architectures interconnecting cancer-related pathways and clinical outcomes.

The knowledge obtained in this study could be useful for a better understanding of BC tumorigenesis and tumor progression as well as for novel, optimized pharmaceutical strategy development.

## RESULTS

### Workflow of our study

**Figure 1:**
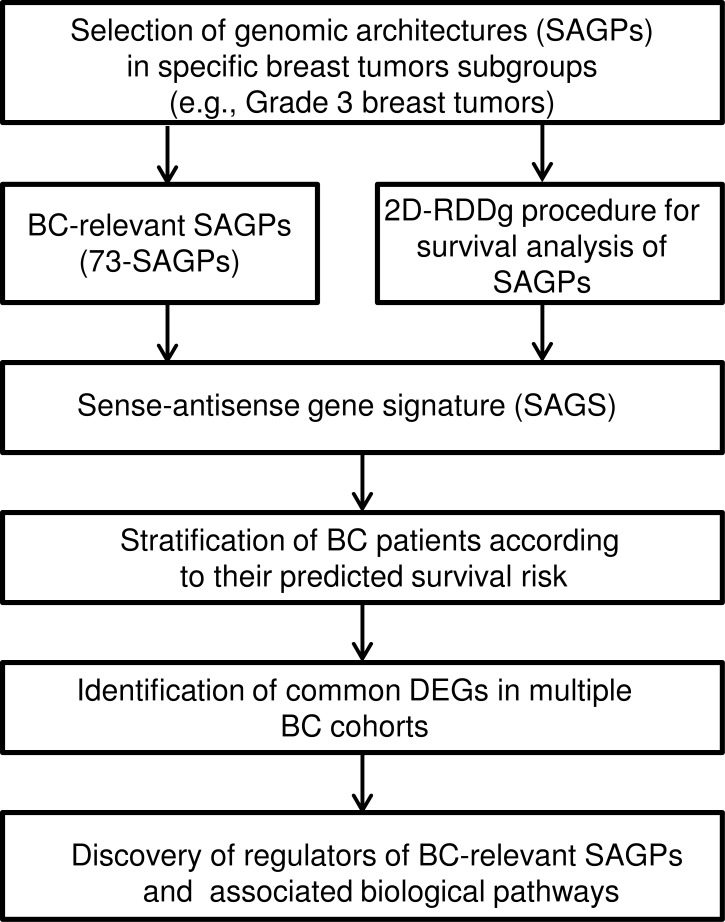
Workflow of the study

The workflow shown in Figure 1 specifies our genomic architecture-centered approach to the genome-wide analysis of the expression patterns of physically associated genes composing SAGPs. Firstly, we focused on the analysis of the well-annotated protein coding-coding SAGPs [5]. Secondly, we analyzed the gene pairs based on their expression levels and correlations of the expressed genes for individual SAGPs. The functional characteristics of such gene pairs were studied here for the identification of possible regulatory molecular mechanisms of BC development, associated with the expression patterns of SAGPs.

Thirdly, we selected the 73 BC-relevant SAGPs set (73-SAGPs), for which: i) the expression values of gene partners in a given SAGP were significantly correlated in the histologic grade 3 breast tumor datasets and ii) the differences of the distributions of correlation coefficients between gene partners of the SAGPs were significantly associated with the differences in the pathobiological status of breast tissue samples (e.g., normal vs. tumor) and clinical outcomes of BC patients.

Among the 73-SAGPs, we identified the most significant prognostic paired gene sub-set, termed SA gene signature (SAGS), which reliably dichotomized the patients into low-risk (LR) and high-risk (HR) cancer development subgroups. SAGS-based stratification was followed by differentially expressed genes (DEGs) and functional annotation and gene ontology (FA/GO) enrichment analyses, providing the finding of specific biological processes, pathways and genes associated with BC patient clinical outcome stratification.

Potential transcriptional drivers of the BC-relevant 73-SAGPs and the DEGs of the specified pathways were selected after Transcription Factor (TF) Binding Motifs and ChIP-seq data analyses.

### Identification of BC-relevant SAGPs and their characteristics within G3 breast tumors

To reduce the candidate list of clinically relevant SAGPs, we aimed to identify a subset of SAGPs with concordant expression of their gene partners. Using the criteria of identification of BC-relevant SAGPs (Supplementary file 2: Methods and Analyses), we retrieved 728 non-redundant protein coding-coding SAGPs, represented by 1383 gene symbol IDs from the USAGP database [5](the USAGP database supports hg18 NCBI assembly). Among these SAGPs, 334 non-redundant SAGPs whose gene partners mRNA expression was supported by at least one high-quality Affymetrix U133A&B probe set presented in the USAGP database (Supplementary file 1: Table S1A). Next, we focused on the identification of the expression patterns of SAGP gene partners within histological Grade 3 (G3) tumors. G3 tumors represent a genetically distinct tumor class, characterized by highly aggressive behavior, frequent metastases, drug resistance and poor disease outcome [27]. We proposed that a selection of significantly correlated SAGPs in this tumor class could help us to elucidate the associations of co-expressed SAGPs with disease outcome and pathobiological features in BC.

According to the current molecular classification, breast cancers are classified into five intrinsic subtypes: normal-like, luminal A, luminal B, *ERBB2/HER2* “positive” and basal-like. G3 tumors are heterogeneous and comprise mostly the luminal B, *ERBB2/HER2* “positive” and basal-like subtypes. The basal-like subtype is a highly aggressive carcinoma that is often resistant to chemo- and hormonal therapy and has an increased occurrence in patients with germline BRCA1 mutations or in patients of African ancestry [28]. The basal-like BC subtype, also known as predominantly “triple negative” BC, often lacks the expression of estrogen, progesterone and *HER2* receptors. Because G3 basal-like tumors represent the most challenging BC subgroup with respect to chemo- and hormonal post-surgery therapy and clinical outcome prediction, we also investigated the SAGPs as discriminative biomarkers of basal-like tumors.

In this context, we considered the G3 basal-like breast tumors and the rest of the G3 tumors as two G3 subgroups. The subgroup of the G3 “non-basal-like” tumor samples was represented by *ERBB2/HER2*”positive”, luminal B, luminal A and “normal-like” subtypes [29] (Supplementary file 2: Methods and Analyses, Supplementary file 3: Figure S1A). The intrinsic tumor subtypes classification annotation has been used according to the information available in the original data sets (Supplementary file 2: Table S11). Screening of significantly correlated SAGPs (Kendall's Tau correlation, *p* < 0.05) within G3 basal-like tumors in three independent patient cohorts revealed that 40 correlated SAGPs were common across these 3 cohorts (Supplementary file 3: Figure S1B and Supplementary file 1: Table S1B). The gene partners in each of the 40 pairs had positive correlation coefficient values (Supplementary file 1: Table S1B). We also identified 52 significantly and positively correlated SAGPs in the non-basal-like tumor subgroup. A total of 21 of the 40 SAGPs were significantly correlated in the G3 basal-like tumor samples, but not in the G3 non-basal like tumor samples (Supplementary file 1: Table S1C). Among the 42 genes of the 21 SAGPs, the DEG analysis identified 14 significant genes, discriminating the G3 basal-like from the G3 non-basal-like tumor samples (t-test; Q-value < 0.05, Supplementary file 3: Figure S1C). Among the genes significantly co-activated in G3 basal-like tumor samples, we identified 3 reproducible and concordantly up-regulated SAGPs (*ABI1/PDSS1*, *DIS3/BORA* and *WDR77/ATP5F1*). These pairs could be considered as promising tumor subtype-specific, up-regulated biomarkers of G3 basal-like breast tumors. These pairs could have an advantage over the “down-regulated” biomarkers used in clinical studies for the identification of basal-like BC subtype [30].

In total, using three independent BC cohorts, we identified 73 SAGPs (73-SAGPs set) where the expression levels of both genes in a given SAGP were significantly correlated within the G3 basal-like and/or G3 non-basal-like breast tumors. Hypergeometric test revealed a high frequency of co-occurrence of significantly correlated SAGPs, co-expressed in two studied data sets of G3 patients (Supplementary file 3: Figure S1B). This indicates that the 73 selected, positively correlated SAGPs could be involved in the same or interconnected gene regulatory pathways and/or networks in the cells of G3 BC tumors.

### Discriminative characteristics of 73-SAGPs between breast tumors and normal tissues and between BC histologic grades

We further investigated whether the gene expression correlation pattern of 73-SAGPs could reflect certain essential BC genetically/clinically distinctive features, such as differences between normal and tumor breast tissues or between breast tumor grades.

**Figure 2:**
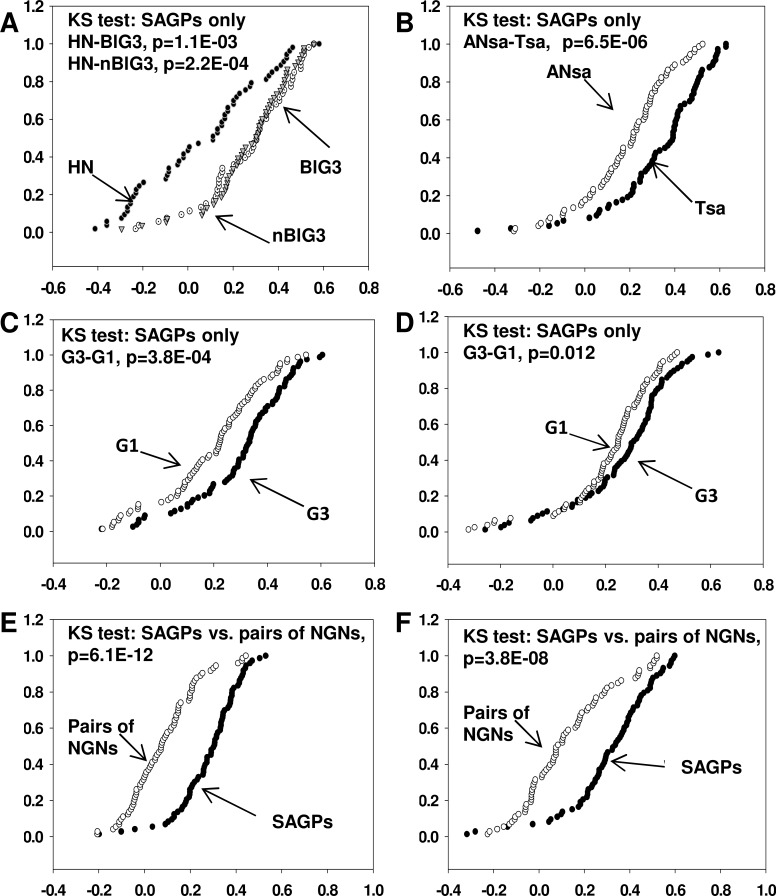
Comparison of cumulative curves of Kendall's Tau correlation coefficients in various gene sets For each SAGP, one corresponding representative pair of Affymetrix probe sets with the strongest Kendall's Tau correlation coefficient (positive or negative) was selected. X axis: Kendall's Tau correlation coefficient, Y axis: cumulative relative frequency. **A.** Cumulative curves for 53 SAGPs in grade 3 “basal-like” (BlG3, white circles), grade 3 “non-basal-like” (nBlG3, light gray triangles) from the Uppsala BC cohort and histologically normal breast epithelium samples (HN, black circles). **B.** Cumulative curves for 73-SAGPs (Tsa) in 30 breast tumors (black circles) and 62 histologically normal epithelium samples located adjacent to tumors (Ansa, white circles). Cumulative curves for G3 and G1 BC patients obtained from the Uppsala **C.** and Stockholm **D.** cohorts. Black circles represent the clinical subgroup with G3 tumors (*n* = 55 for Uppsala cohort; *n* = 61 for Stockholm cohort), and white circles correspond to the clinical group with G1 tumors (*n* = 68 for Uppsala cohort, *n* = 28 for Stockholm cohort). Cumulative curves for all BC patients in the Uppsala BC cohort **E.** (*n* = 249) and the set of 38 BC cell lines **F.** [32]. Black circles represent the group of 73-SAGPs, and white circles represent the group of 73 NGNs.

We compared the cumulative frequency distribution functions of the Kendall's Tau correlation coefficient values estimated between the gene partners of the 73-SAGPs in breast tumors and normal tissues (Figure 2A and 2B and Supplementary file 3: Figure S1D). There were no significant differences between G3 basal-like and G3 non-basal-like breast tumors or between the two normal breast tissues used as negative controls (p>0.05, KS test) (Figure 2A and Supplementary file 2: Table S2). However, a highly significant systematic shift of the correlation value distributions was evident for almost all BC tissues compared to normal tissues (Figure 2A and 2B, Supplementary file 2: Table S2).

The Kolmogorov-Smirnov (KS) tests demonstrated significant differences between the Kendall's Tau correlation coefficient values in high grade (G3) compared to low grade (G1) breast tumors (Figure 2C and 2D) in the 73-SAGPs. Taken together, the correlation coefficient values between the *in-cis* gene partners of the 73-SAGPs are significantly associated with different pathobiological states of breast tissue (for instance normal vs. BC, G3 vs. G1 tumors). Therefore, as a co-activated functional gene subset, they might be involved in deregulated pathobiological gene networks and pathways in BC. Next, we addressed the question of which molecular mechanisms could be considered as potential regulators of the 73-SAGPs in BC.

### DNA CNVs is not a major factor of positive correlations between the gene partners of the 73-SAGPs

Many pathological disturbances and abnormal correlations between genes in cancers are due to their co-localization within the same DNA amplicons [31]. We hypothesized that the significant and positive correlations between the gene partners of the 73-SAGPs are specific and are not due to DNA amplifications in the SAGPs loci. To test this hypothesis, we selected 73 pairs of the nearest gene-neighbors (NGNs) of the 73-SAGPs. The criteria for selection of the NGNs are stated in Supplementary file 2: Methods and Analyses and Supplementary file 1: Table S3A.

CNVs often cover mega-base regions in the human genome. Therefore, we expected that an SAGP and its corresponding NGN pair could be located in the same CNV region. Indeed, CNVs for NGNs were very similar to those of corresponding co-localized 73-SAGPs (Supplementary file 3: Figure S2). Wilcoxon matched pairs test revealed no significant differences in the CNVs between gene sets of the co-localised NGNs and 73-SAGPs studied in two independent data sets (Supplementary file 1: Tables S4A-S4C, Methods).

In contrast, the Kendall's Tau correlation coefficients for “in-cis” pairs of co-localized NGNs were significantly lower than the correlation coefficients for the “in-cis” gene partners of the 73-SAGPs analyzed in the set of 38 BC cell lines [32] as well as in another primary breast tumor cohort (Figure 2E and 2F, Supplementary file 1, Table S3B). These results suggest that CNV are not a major factor in the positive correlation between the gene partners of the 73-SAGPs.

### Identification of survival significant SAGPs

Because the 73-SAGPs demonstrated significant associations with distinct BC pathological states and subgroups, we hypothesized that they could be involved in specific deregulated pathobiological pathways. Survival prediction analysis can be used to investigate whether a gene set contains genes and/or gene pairs significantly associated with distinct survival/pathological outcomes [33, 34]. Pathologically relevant gene signatures in turn can be utilized for in-depth characterization of deregulated oncogenic pathways and the discovery of potential drug targets in cancer [33, 35-37].

Because gene pairs in 73-SAGPs are significantly correlated, we assumed that a survival-significant SAGP with a synergistic effect on a patient survival compared to individual genes could be preferentially utilized as a distinct stratification feature for survival prediction analysis [25]. In this study we use the survival prediction method called data driven grouping (DDg) method (Materials and Methods; [25], [29, 34]) based on Cox proportional hazards model and the selection procedure using the optimal patient statistical partition models (Supplementary file 2: Definitions) applied for every SAGP. The most significant patient statistical partition model is defined based on the cutoff values for both gene expression values, each of which maximizes a separation the relatively LR and HR prognostic groups.

We assessed the survival significance of the 73-SAGPs using our previously developed, 1-D DDg, 2-D DDg and a novel 2-D RDDg procedures (Supplementary file 3: Figure S5) [34].

**Figure 3:**
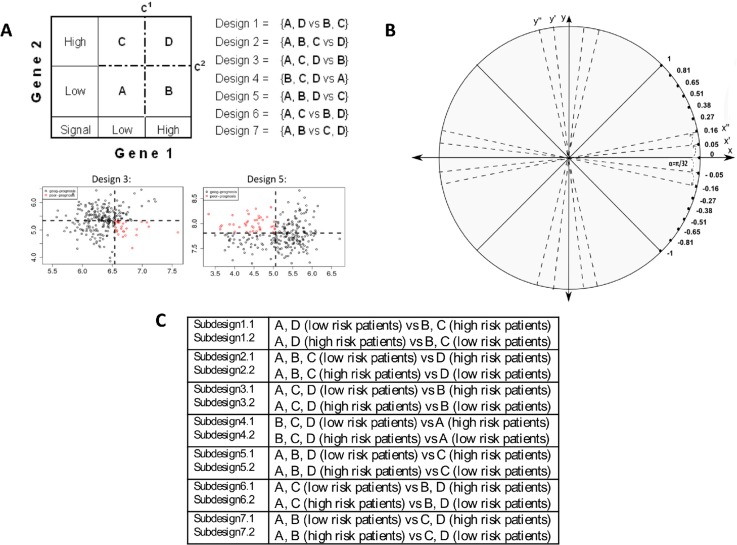
2-D RDDg: important components of the survival prediction method **A.** Grouping of gene pairs (genes 1 and 2 with respective cut-offs c^1^ and c^2^) and all possible two-group designs (Designs 1-7) used in both the 2-D DDg and 2-D RDDg [29, 34]. Red circles mark the sector of high risk of recurrence and black circles mark the sector of low risk of recurrence. **B.** The advanced model of gene-pair grouping using an additional step specific for the 2-D RDDg that involves the iterative rotation of axes X and Y without losing their orthogonality. **C.** Patients partition sub-designs.

**Figure 4:**
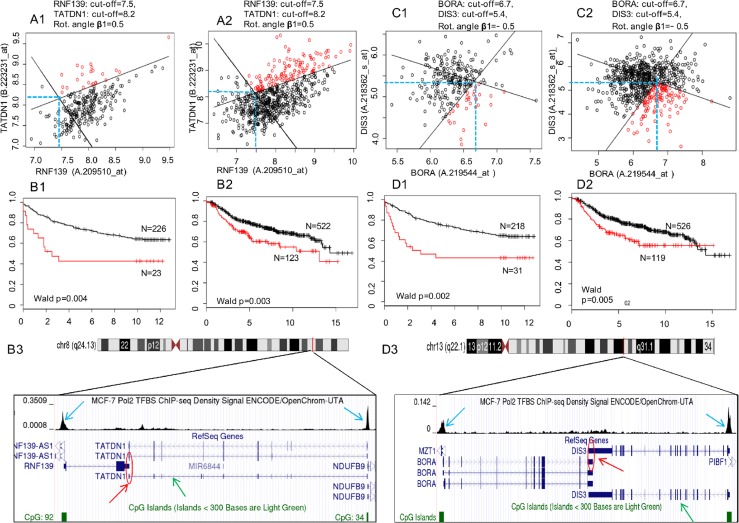
Survival prediction analysis using the 2-D RDDg for two SAGPs from the SAGS **A1., B1., C1.** and **D1.** Uppsala cohort (training). **A2., B2., C2.** and **D2.** Metadata cohort (testing). **A1., A2. and C1., C2.** Optimal partitions of expression domains in BC patients using expression values for two pairs of Affymetrix probe sets (each pair corresponds to two host genes in a SAGP) (see Materials and Methods section). Black solid rotated lines correspond to the horizontal (X) and vertical (Y) lines (blue dashed lines) for optimal gene expression cut-offs. Partition parameters (design, gene expression cut-offs and rotation angle) were fixed in the training groups and reproduced in the testing groups. **B1., B2.** and **D1., D2.** Differences between Kaplan-Meier survival curves for the LR and HR subgroups obtained after the patients partitioning within each studied cohort. X axis: disease-free survival (DFS), years; Y axis: survival probability. Black circles and survival curves indicate the LR prognosis group, and red circles indicate the HR prognosis group. Parts of the panel with the same letter correspond to the same SAGP. **B3.** and **D3.** The same two SAGPs visualised in the UCSC Genome Browser [87]. Red arrows represent the sense gene partners, green arrows represent the antisense gene partners and red circles represent the regions of SA overlap in the SAGPs. The enriched ChIP-seq regions for Pol2 of high read density in the ChIP-seq experiment relative to total input chromatin control reads (according to ENCODE project, blue arrows) indicate that the gene promoters in the SAGPs are active in MCF-7 breast cancer cells.

The 2-D RDDg procedure was developed to obtain less biased and more accurate patients stratification in SAGPs compared to 2-D DDg (see Materials and Methods, Supplementary file 2: Methods and Analyses). The 2-D RDDg is a prognostic method of patient risk of disease development stratification and the feature selection based on generalization of the 2-D DDg method [34] (Figures 3 and 4). This method further refines patient partitioning by adjusting the rotation of the horizontal and vertical axes to yield a more optimal separation of the patient survival curves (Supplementary file 2: Methods and Analyses and Figure 3) than 2-D DDg. Similar to the 2-D DDg, the improved 2-D RDDg allows for the stepwise selection of synergistic survival-significant SAGPs.

Screening of the 73-SAGPs using the 2-D RDDg in the Uppsala and Stockholm cohorts identified twelve synergistic SAGPs (Table 2) that passed our criteria for significance (Wald *p*-value < 0.05; common design of 2-D partition, the same gene expression cutoff values and the same rotation angles) in both cohorts (Supplementary file 1: Table S8) and were collectively termed the sense-antisense gene signature (SAGS). Survival prediction analysis (Supplementary file 1: Table S5) and literature analysis (Supplementary file 2: Tables S6 and S7) of the genes composing the 73-SAGPs and SAGS provide evidence of the association of the studied genes with cancer and suggest a possibility for their therapeutic targeting.

**Table 2:** Host genes, Affymetrix probe sets and representative RNA transcripts for SAGS

#SAGP in the SAGS	Host gene symbol	Affymetrix probe set ID	Best RNA ID1	DNA strand	Host gene description (UCSC genome browser)	Chromosome band
1	*C18orf8*	B.232348_at	CA395475*	-	Colon cancer-associated protein Mic1	18q11.2
	*NPC1*	A.202679_at	NM_000271	-	Niemann-Pick disease, type C1 precursor	
2	*BORA*	A.219544_at	NM_024808	+	Bora, aurora kinase A activator	13q22.1
	*DIS3*	A.218362_s_at	NM_001128226	-	DIS3 mitotic control homolog (S. cerevisiae)	
3	*AIMP2*	A.209971_x_at	NM_006303	+	Aminoacyl tRNA synthetase complex-interacting multifunctional protein 2	7p22
	*EIF2AK1*	A.217736_s_at	NM_014413	-	Eukaryotic translation initiation factor 2-alpha kinase 1	
4	*SHMT1*	A.217304_at	Y14488**	-	Serine hydroxymethyltransferase 1 (soluble)	17p11.2
	*SMCR8*	B.227304_at	NM_144775	+	Smith-Magenis syndrome chromosome region	
5	*DOK4*	A.209690_s_at	NM_018110	-	Docking protein 4	16q21
	*POLR2C*	A.208996_s_at	NM_032940	+	DNA directed RNA polymerase II polypeptide C	
6	*MRPS18C*	B.228019_s_at	NM_016067	+	Mitochondrial ribosomal protein S18C	4q21.23
	*FAM175A*	B.226521_s_at	NM_139076	-	Family with sequence similarity 175	
7	*CTNS*	A.204925_at	NM_001031681	+	Cystinosin, lysosomal cystine transporter	17p13
	*TAX1BP3*	A.209154_at	NM_014604	-	Tax1 (human T-cell leukaemia virus type I) binding protein 3	
8	*EME1*	B.234464_s_at	NM_001166131	+	Essential meiotic endonuclease 1 homolog 1	17q21.33
	*LRRC59*	B.234812_at	HY246925***	-	Leucine rich repeat containing 59	
9	*VPRBP*	B.226481_at	BC022792****	-	Vpr (HIV-1) binding protein (VPRBP)	3p21.2
	*RBM15B*	A.202689_at	NM_013286	+	RNA binding motif protein 15B	
10	*RNF139*	A.209510_at	NM_007218	+	Ring finger protein 139	8q24.13
	*TATDN1*	B.223231_at	NM_001146160	-	TatD DNase domain containing 1	
11	*SSB*	A.201139_s_at	NM_003142	+	Sjogren syndrome antigen B	2q31.1
	*METTL5*	A.221570_s_at	NM_014168	-	Methyltransferase like 5	
12	*BIVM*	B.222761_at	NM_001159596	+	Basic, immunoglobulin-like variable motif	13q33.1
	*KDELC1*	A.219479_at	NM_024089	-	KDEL (Lys-Asp-Glu-Leu) containing 1	

The best RNA IDs for the corresponding Affymetrix probe sets were chosen. The priority selection criteria were defined as follows: a) best ID by chromosome coordinates, b) well-characterised RefSeq NM IDs, c) RefSeq mRNA IDs, and d) EST (expressed sequence tags) IDs.

* paired transcript located on the same strand as the NPC1 gene within the territory of the C18orf8 gene;

** mRNA sequence located within the territory of the SHMT1 host gene isolated from the clone, pUS1215 (BC cell line ZR-75-1, UCSC Genome Browser);

*** EST sequence isolated from the clone, H05D007L23(LIBEST_027875 RIKEN full-length enriched human thymus cDNA library, UCSC Genome Browser), transcript presumably belongs to the LRRC59 host gene;

**** mRNA from the cDNA clone, MGC:23092IMAGE:4853730 (NIH_MGC_48 library, UCSC Genome Browser). Pairs of Affymetrix probe sets #1, #4 and #8 were included in the SAGS because their best representative pairs of transcripts belong to the pairs of host genes with sense-antisense overlaps, and they satisfy the criteria of survival significance and synergism in two independent cohorts (Supplementary file 1: Table S8).

### Identification of LR and HR BC patient subgroups associated with SAGPs

**Figure 5:**
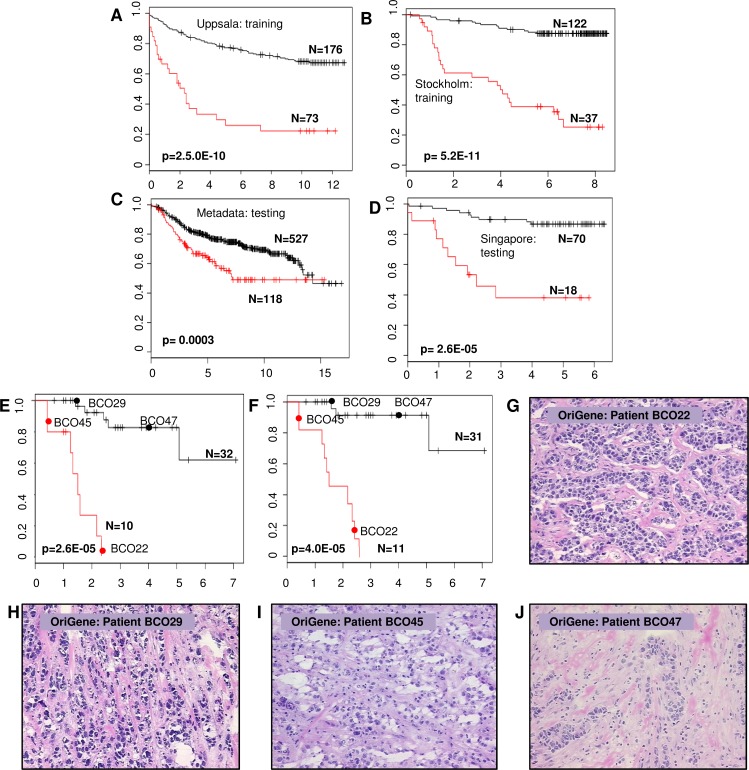
Kaplan-Meier survival prediction analysis in BC patients using SAGS Survival differences between LR and HR subgroups of BC patients after applying the full SAGS in each BC patient group. X axis: DFS, years; Y axis: survival probability. **A.** and **B.** The Uppsala and Stockholm cohorts, respectively; **C.** and **D.** Cross-cohort validation in the Metadata and Singapore cohorts, respectively; **E.** and **F.** qRT-PCR reproducibility of the expression microarray-derived SAGS. Forty-two BC patients from the OriGene cohort were stratified using only nine SAGPs from the SAGS and the U133-Plus microarray platform for gene expression detection **E.**; the same 42 BC patients from the OriGene cohort were stratified using the same nine SAGPs (eighteen genes) and utilising the strand-specific qRT-PCR **F.**. The red circles and the black circles indicate the survival curve locations of patients whose tumor tissue sections (hematoxylin-eosin, 20x) are shown in (G - J); tissue sections shown in **G.** and **I.** (HR subgroups) belong to patients with G3 tumors; tumor tissue sections shown in **H.** and **J.** (LR subgroups) also belong to patients with G3 tumors (Supplementary Table S12).

SAGS was further used to stratify the Uppsala and Stockholm BC cohorts using the gene pairs weighted voting grouping procedure (WVG, see Methods, Figure 5A and 5B) [38]. The WVG procedure (Supplementary file 2: Methods and Analyses) combines the information from statistical partition models for each individual SAGP obtained using either the 2-D DDg or the 2-D RDDg into more integrated and significant patient partitions.

We also compared the performance of the 2-D RDDg with that of the 2-D DDg in the Stockholm cohort. The 2-D RDDg (Supplementary file 3: Figure S7) provided more accurate (lower Wald *p*-value) patient stratification for many of the analyzed SAGPs. Therefore, the 2-D RDDg has an advantage over the 2-D DDg for more accurate prediction of patient subgroups based on clinical outcomes.

To ensure the robustness for identification of pathological pathways associated with SAGPs, we performed SAGS-based stratification in 2 additional BC datasets (Metadata and Singapore) (Table 1). To ensure the cross-cohort reproducibility of the SAGS stratification, optimal stratification parameters of SAGS identified in the Uppsala and Stockholm cohorts (design, rotation angle and two gene expression cutoffs) were fixed and applied in the Metadata and Singapore datasets. Applying the WVG procedure after the 2-D RDDg using the SAGS allowed us to significantly stratify (WVG Wald *p* < 0.01) patients from the studied cohorts into low-risk (LR) and high-risk (HR) subgroups (Figure 5A - 5D: Uppsala (*p* = 2.5E-10), Stockholm (*p* = 5.2E-11), Metadata (*p* = 3.0E-04) and Singapore (*p* = 2.6E-05). The AUCs from the ROC analysis were significant in all of the studied cohorts: Uppsala (*p* < 0.0001), Stockholm (*p* < 0.0001), Metadata (*p* < 0.001) and Singapore (*p* < 0.0001). The prognostic accuracy varied from 67.9% (Metadata) to 86.0% (Stockholm) (Supplementary file 3: Figure S8).

**Table 1:** Groups and cohorts of BC patients used to verify cross-cohort reproducibility of the SAGS using the 2-D RDDg coupled with the WVG procedure

Training cohorts (num. of patients)	Ref. in the current report	Cross-validation cohorts (num. of patients)	Ref. in the current report	Literature ref.
1.Uppsala (249)*	Figure 5A	1.Metadata cohort (645): combined Oxford& Guys Hospital, Harvard 2, Marseille and BII-OriGene cohorts.	Figure 5C	[27, 71, 73, 88]
2.Stockholm (159)*	Figure 5B	2.Singapore (88)	Figure 5D	[27]

* training was performed in both cohorts independently; the best training parameters common for both cohorts (gene expression cut-offs, partition designs and rotation angles) for each gene pair have been fixed and applied in the testing cohorts.

The cross-platform reproducibility of the microarray-driven SAGS was assessed using qRT-PCR (see Methods) in the BII-Origene cohort. We used the microfluidic high-throughput Fluidigm technology (Fluidigm, San Francisco, CA) for rapid, accurate and simultaneous detection of the expression of multiple genes. The SAGS stratification in the same 42 BC patients using either microarray or qRT-PCR gene expression data showed strong concordance with the patient partitioning into LR and HR subgroups (Cohen's Kappa = 0.56, *p* = 0.001) (Figure 5F and 5G). Images of frozen tumor tissue sections (OriGene Technologies, predominantly G3 tumors) were independently verified by a pathologist from Singapore (Supplementary file 1: Table S12). Using our method, tumors were reclassified into 2 distinct clinical subgroups (Figure 5F - 5K).

Taken together, the SAGS patients stratification, as an important intermediate step in our study workflow (Figure 1), resulted in the identification of reproducible, clinically distinct BC patient subgroups associated with SAGPs.

### Proteasome and precatalytic spliceosome genes are significantly over-expressed and over-represented in HR patient subgroups identified by the SAGS

We assumed that SAGS-based patients survival stratification reflects some fundamental patho-biological properties and pathways of the BC types of the relatively poor (HR) and good (LR) disease outcome patient subgroups. To test this hypothesis, we identified differentially expressed genes (DEGs) between the subgroups.

DEGs between HR and LR subgroups, defined above by the SAGS, were derived using the EDGE software [39] providing the selection of high confidence FDR-corrected DEGs. We analyzed data of Uppsala, Stockholm and Metadata cohorts and selected several thousand DEGs in each cohort. 1381 genes were common for these three data sets (t-test FDR corrected Q-value < 0.01, Supplementary file 1: Table S9A), suggesting a reproducibility of these DEGs across the patient cohorts.

Noteworthy, this DEG set was highly-enriched with 201 breast tumor aggressiveness grading (TAG) signature genes(Suppl. data in [27]) (118 out of the 201 TAG genes; hypergeometric test, *p* = 44.3E-82, Table S9A), which are mostly involved in the cell cycle, mitosis and cell proliferation [27]. TAG is the microarray-based molecular analogue of the histologic grading classifier of BC, separating the histologic grade 2 (G2) BCs in the histologic grade 1-like (G1-like) and histologic grade 3-like (G3-like) tumor genetic sub-classes. This classifier proposes two major genetically-defined classes of BC defined as low-aggressive (G1+G1-like) and high-aggressive (G3-like+G3) tumor classes with significant difference in clinical outcomes. We also found that the SAGS-stratified LR and HR BC subgroups are significantly correlated with the patient subgroups obtained by the TAG signature [27].

The 71% (978/1381) of DEGs were up-regulated in the HR patient subgroup. Using DAVID bioinformatics software [40], we identified many biologically distinct gene subsets, associated with cancer and its aggressiveness. These gene subsets included the genes enriched under the terms “Proteasome” (*p* = 5.5Ee-17), “Cell cycle” (*p* = 3.3e-14), “DNA replication” (*p* = 2.1e-10) (KEGG_PATHWAY), “DNA repair” (*p* = 1.09E-08) (GOTERM_BP_FAT), “Spliceosome” (8.5e-05), “Pyrimidine metabolism” (*p* = 1.8E-03), “t-RNA biosynthesis” (*p* = 7.7E-03) (KEGG_PATHWAY). 188 genes out of the analyzed 978 genes may encode the proteins containing experimentally defined mutagenesis sites (*p* = 2.4E-13, “mutagenesis site”, UP_SEQ _FEATURE) (Supplementary file 1: Table S9B). Importantly, among the 403 DEGs significantly down-regulated in the HR subgroups, the gene terms associated with cell locomotion, cell adhesion and cell migration were highly enriched (Supplementary file 1: Table S9B).

**Figure 6:**
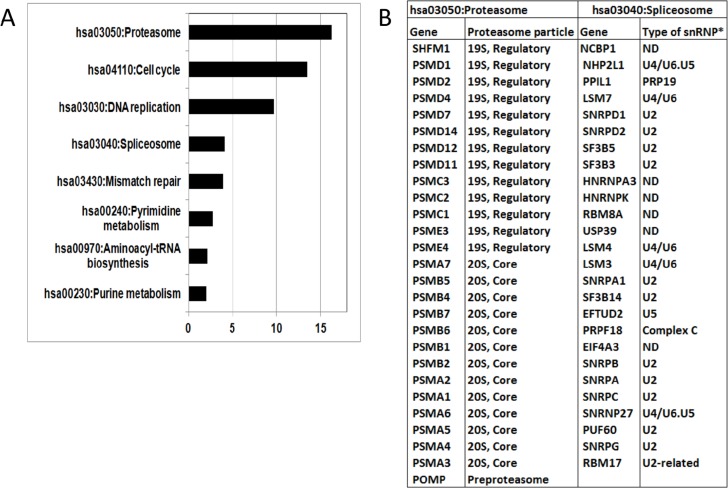
**A.** Biological pathways (KEGG) characterising genes over-expressed in the HR subgroups identified by the SAGS. X-axis: -log10 Bonferroni corrected p-value (DAVID software). **B.** Compositions of proteasomal and spliceosomal genes identified by Gene Ontology analysis. *: snRNP type was determined according to [41]. ND - not determined.

Further analyses of 27 proteasome genes identified under the DAVID term ”hsa03050: Proteasome” revealed that they are evenly representing both the 20S core particle and the 19S regulatory particle of the proteasome (Figure 6B). The 26 genes, listed under the term “hsa03040: Spliceosome” (KEGG_ PATHWAY, Table S9B), predominantly belong to the U2-, U4/U6-snRNPs, including one gene of the *PRP19* complex (Figure 6B). The U1-, U2-snRNPs, the *PRP19* complex and the U4/U5/U6 tri-snRNPs predominantly participate in the same specific stage of the spliceosome cycle, termed *the precatalytic spliceosome, or complex B*. This stage of the spliceosome cycle is followed by the assembly of the catalytic spliceosome, or active complex C, in which the chemical steps of splicing occur. The U4/U6 snRNP is absent in complex C [41]. Interestingly, a literature analysis of the spliceosomal DEGs suggests their potential as highly promising anti-spliceosome drug targets (Supplementary file 2: Methods and Analyses).

Thus, our meta-analysis using the 73-SAGPs and the SAGS identified specific, HR prognostic BC patient subgroups, whose tumors displayed distinct GO characteristics. The profile of DEGs down-regulated in the HR subgroups displayed enrichment in FA/GO terms for cell locomotion, cell adhesion and cell migration.

The profile of DEGs over-expressed in the relatively HR subgroups was significantly enriched for genes involved in the cell cycle, DNA damage, DNA repair and certain deregulated genes of the proteasome and spliceosome.

### GABPA provides a mechanistic link between the 73-SAGPs and certain cell cycle, proteasomal and spliceosomal genes

Next, we studied whether the 73-SAGPs, the proteasomal and spliceosomal genes could be driven by any of the regulatory factors in BC cells. We analyzed the proximal promoters (−450/+50 bp) for the enrichment of transcription factor binding motifs (TFBMs) using the Jaspar database from PSCAN software [42] in the gene sets listed below. As negative controls, we used 3 gene sets: i) the set of 102 genes involved in PNGs (Supplementary file 3: Figures S3 and S4, Supplementary file 1: Table S3C), i.e., the pairs of co-localized and robustly correlated genes in the same BC subgroups as the 73-SAGPs but without SA overlaps; ii) the set of 146 NGNs (Table S3A) and iii) 150 top differentially expressed, down-regulated genes in HR subgroups after the SAGS stratification (DEDR genes set) (Supplementary file 1: Table S9C,Methods and Analyses). The “KEGG genes” set (148 genes) included differentially expressed, significantly upregulated genes (Q-value < 0.01) in HR subgroups classified by the SAGS in the three studied cohorts and enriched under the DAVID category, “KEGG_PATHWAY” (Supplementary file 1: Table S9C).

The gene enrichment analysis revealed the strongest significant enrichments of TFBMs for *ETS*-domain TFs (GABPA, ELK1 and ELK4: Bonferroni corrected *p* = 1.4E-14, *p* = 9.2E-13 and 6.4E-11, respectively) in the promoters of the 73-SAGPs. Less prominent enrichment of TFBMs for GABPA and ELK4 was observed in the NGNs set (*p* = 9.5E-06 and *p* = 2.4E-04), and no enrichment was observed in the PNG set. The motifs for GABPA were also overrepresented in the KEGG genes set (*p* < 0.001), but not in the DEDR gene set. In contrast, ERα, which is involved in BC cells proliferation and the cell cycle in MCF-7 cells via the cyclin D1-CDK4/Rb/E2F1 pathway [43], showed no TFBMs enrichment in the proximal promoters of the studied gene sets.

Because the TFBM for GABPA (Figure 8A) showed the strongest enrichment in the 73-SAGPs, we further tested whether GABPA can preferentially bind to the proximal promoters (−450/+50 bp) in the 73-SAGPs and other gene sets. We observed the enrichment of GABPA ChIP-seq binding regions (CBRs) in the MCF-7 BC cell line for promoters of the same gene sets (compared to PNGs and NGNs, Fisher's exact test, Figure 8B). Significant enrichment of GABPA CBRs in 73-SAGPs was observed regardless of potential sharing bidirectional promoters in divergent SAGPs (“73-SAGPs_unique” vs. “73-SAGPs_all”). This fact suggested certain regulatory advantages of GABPA binding to proximal promoters of 73-SAGPs compared with the co-localised and correlated pairs of genes without SA overlaps.

**Figure 7:**
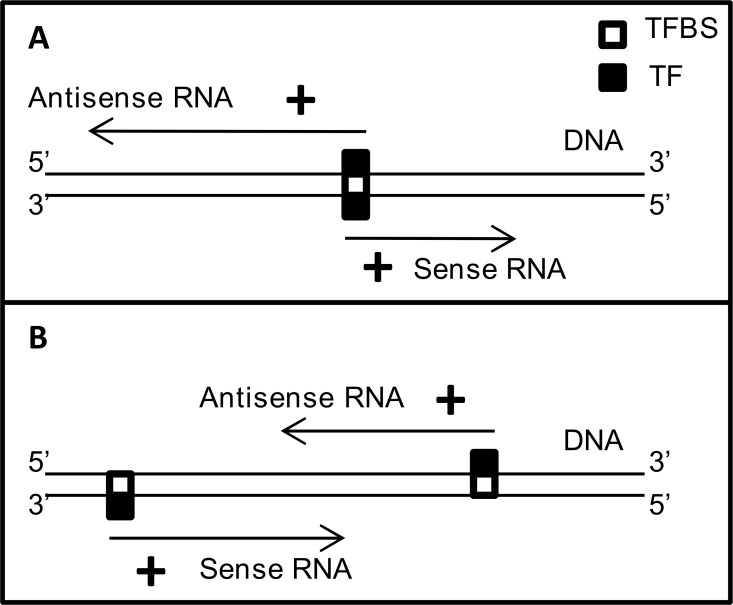
Possible mechanistic models for co-regulation of gene partners in 73-SAGPs **A.** bidirectional transcription via the same TF can lead to positive correlations between gene partners in divergent SAGPs; **B.** transcriptional coordination of gene partners in convergent SAGPs via the same TF.

**Figure 8:**
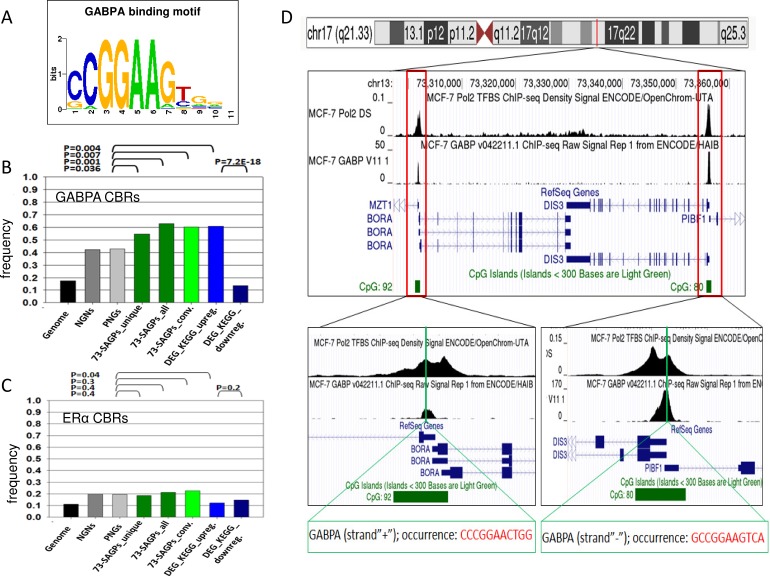
GABPA CBRs in the vicinity of proximal promoters of 73-SAGPs and other gene sets **A.** TFBM for GABPA identified using PSCAN software [42]. **B.** and **C.** Frequencies of CBRs for GABPA and ERα in MCF-7 cells overlapping with proximal promoters in various gene sets; X-axis: various gene sets, Y-axis: the frequency of the number of CBRs. Differences in the frequencies of the occurrence of CBRs overlapping the proximal promoters were assessed using Fisher's exact test. “73-SAGPs_unique”: the frequency of only unique overlaps of proximal promoters with CBRs; “73-SAGPs_all”: for divergent 73-SAGPs within the set, the occurrence of the same unique overlapping CBRs in bidirectional promoters was multiplied by 2 because the TF GABPA can regulate gene expression in the opposite directions [53]; “73-SAGPs_conv”: the subset of convergent 73-SAGPs. “DEG_KEGG_upreg.”: differentially expressed, significantly up-regulated genes (Q-value < 0.01) in the HR subgroups of the three studied cohorts classified by the SAGS; only the genes enriched under the category “KEGG_PATHWAY”(DAVID, Bonferroni *p*-value < 0.05) were analysed; “DEG_top_downreg.”: the top differentially expressed and significantly down-regulated genes (DEDR gene set) (Q-value < 0.01) in the HR subgroups of the three studied cohorts classified by the SAGS. **D.** CBRs for *Pol2* and *GABPA* are co-localised in the proximal promoters of both gene partners in the convergent SAGP *DIS3/BORA* (MCF-7 cells). We used metadata tracks for Pol2 and GABPA from the UCSC Genomic Browser (hg19 assembly) [87]. GABPA TFBMs located within GABPA CBRs were identified using the TF ChIP-seq track from ENCODE with Factorbook motifs (Release 4, February 2014). All peaks highlighted in red indicate the enriched regions of high read density in the ChIP-seq experiment relative to total input chromatin control reads according to the ENCODE project.

Similarly, significant enrichment of GABPA CBRs in the promoters of the KEGG genes (including certain cell cycle, spliceosomal and proteasomal genes) was detected; however, this was not true for ERα CBRs (Figure 8C). Additionally, dramatically lower frequencies of GABPA CBRs in the DEDR genes supported a regulatory role of this TF in the 73-SAGPs and in the KEGG genes set (Figure 8B). Fourteen individual genes and five distinct SAGPs from the SAGS have overlaps of GABPA CBRs with their proximal promoters in MCF-7 cells (Supplementary file 1: Table S13).

Knockdown experiments of GABPA in MCF-7 BC cells in eleven randomly selected convergent 73-SAGPs (twenty two genes) with GABPA CBRs in their proximal promoters (Supplementary file 1: Table S13) revealed down-regulation of both gene partners in 9 cases. We also observed downregulation in 5 out of 6 spliceosomal and in 3 out of 6 proteasomal genes with GABPA CBR/proximal promoter overlaps (Supplementary file 3: Figure S10). This result confirmed that GABPA can be a direct transcriptional co-regulator of its predicted gene-effectors.

## DISCUSSION

This study represents one of a few studies [11-14, 25] aimed at the systematic investigation of the expression patterns of SAGPs in the context of cancer heterogeneity and pathological pathways for better understanding their impact in tumorigenesis and tumor progression, and for optimized pharmaceutical strategies development.

We started with the correlation analysis of SAGPs within the class of histologic G3 breast tumors, because these tumors are highly aggressive and commonly develop drug resistance and a spread of distant metastases. Within the G3 tumor class, the basal-like tumors comprise the most challenging subgroup with respect to post-surgery therapy. To test whether SAGPs can be used as discriminative biomarkers of G3 basal-like tumors, we combined correlation and DEG analyses and identified SAGPs discriminating the G3 basal-like tumors from the rest of G3 tumors, which we termed “non-basal-like”. From 21 subtype-specific SAGPs positively correlated in the G3 basal-like BC subgroup, we selected 3 concordantly upregulated SAGPs. The gene pairs *ABI1/PDSS1*, *DIS3/BORA* and *WDR77/ATP5F1* and their products could be considered as promising up-regulated discriminative biomarkers of G3 basal-like tumors, although further experimental and clinical validation will be needed.

Next, we investigated whether the SAGPs with positively correlated gene partners within G3 breast tumors (73-SAGPs) are associated with tumor initiation and/or aggressiveness. Further analyses revealed that the 73-SAGPs displayed significantly different correlation profiles in BC samples compared to normal breast tissue samples (Figure 2), indicating that they are relevant to tumor initiation and malignancy. An overall systematic positive shift of the correlation coefficient values calculated between the gene partners in the 73-SAGPs was observed in more aggressive tumors (G3) compared to less aggressive tumors (G1). These findings suggest the relevance of 73-SAGPs to tumor aggressiveness and the disease clinical outcome. Several previous studies suggested that the expression patterns of SAGPs demonstrate cancer type- and subtype-specific expression patterns and might be important for further clinical and pharmaceutical implementations [5, 11-14, 20, 21].

Survival analysis and pathologically relevant gene signatures are useful for the characterization of deregulated oncogenic pathways and identification of potential drug targets. Because the identified 73 BC-relevant SAGPs could be involved in tumor initiation and aggressiveness, we studied their association with BC patient survival and applied feature selection methods based on the data-driven grouping prognosis strategy [29, 34, 37].

We also developed a novel 2-D RDDg prognostic method adapted for the refined survival analysis of correlated gene pairs (including the 73-SAGPs). Our screening workflow combining the 1-D DDg, 2-D DDg, 2-D RDDg and WVG procedures [29] (see Methods, Supplementary file 3: Figure S5) resulted in the identification of the pathologically relevant SAGS. Twelve of the twenty-four genes comprising the SAGS (and used for construction of our SAGS) are reportedly associated with various cancers. Seven genes (*BORA*, *DIS3*, *POLR2C*, *FAM175A*, *EME1*, *RNF139* and *SHMT1)* are considered potential biomarkers and/or potential targets for radiotherapy and chemotherapy, as well as cancer susceptibility, cancer progression and metastasis-related genes (Supplementary file 2: Table S7). SAGS was applied and tested using large cancer microarray datasets that encompassed eight independent BC cohorts (1161 tumors in total). SAGS significantly and reproducibly stratified all breast tumors into LR and HR subgroups.

Next, our DEG and FA/GO comparative analyses between LR and HR subgroups derived by SAGS, revealed several crucial tumorigenic processes and molecular functions associated with 73-SAGPs expression that might be useful for future discovery of novel prognostic biomarkers and therapeutic targets. In particular, proteasome- and precatalytic spliceosome-specific genes were enriched in the HR subgroups of the studied BC cohorts. Several reports indicate that antisense transcription and alternative splicing are tightly and mechanistically coordinated processes [2, 22]. Alternatively, *PRP19* complex, a key element of precatalytic spliceosome [41], is also known as an important regulator of proteasome degradation [44]. Therefore, here we found functional links of SA transcription with splicing and proteasome degradation which might reflect the important inter-pathway connections regulating the BC progression.

Although predominantly positive correlation profiles of SA transcripts/gene pairs in cancers were previously reported [5, 22], the exact molecular mechanisms underlying this phenomenon remain unclear. Abnormal positive correlations among many genes in cancers could be due to their co-localization within the same DNA amplicons and equivalent DNA CNV changes [31]. Additionally, the specific molecular mechanisms coordinating the expression of SA transcripts described in the literature include i) the use of shared regulatory regions for common TFs [45]; ii) chromatin activation in SA overlapping regions, such as antisense-RNA-mediated DNA demethylation [46]; iii) stabilization of a sense transcript by its antisense transcript [7, 8]; iv) selective alternative pre-mRNA maturation [22, 47]. In this report we investigated two first mechanisms in more detail.

Because DNA CNV might be not a substantial coregulatory factor of the 73-SAGPs (see Results), we further investigated whether certain TFs are the potential regulators of the 73-SAGPs. The proximal promoters of the 73-SAGPs were significantly enriched in TFBMs and CBRs for GABPA (Figure 8) compared to the negative control sets of paired genes without sense-antisense overlaps (NGNs and PNGs). Knockdown of GABPA in MCF-7 cells confirmed its direct regulatory role of the 73-SAGPs in BC cells (Supplementary file 3: Figure S10).

GABP is necessary and sufficient for quiescent cells to re-enter the S phase of the cell cycle, independent of D-type cyclins, CDKs and E2Fs [48]. Several pieces of evidence support the idea that *GABPA* is an important gene in the regulation of lineage-restricted genes [49] and stem cell renewal and differentiation [50, 51].

Here we identified the specific GABPA-dependent gene network, which includes not only divergent (head-to-head configuration) but also convergent 73-SAGPs (tail-to-tail configuration) [5] (Figure 8B). Although we focused on GABPA, several other ETS*-*domain factors (e.g., PEA3, ERM, ETS-1, ETS-2 and ESE-1 [52]) are not excluded as alternative regulators for many genes of the network during BC progression.

In context of the GABPA gene network, we can suggest two models of transcriptional coordination of gene partners in the BC-relevant SAGPs. The first model incorporates the known mechanism of sharing common TF in bidirectional promoters of divergent SAGPs [19] because it was shown that GABPA can regulate bidirectional transcription [53] (Figure 7A). The second model implies parallel coordination of the expression of SA transcripts in a convergent SAGP via the same TF (Figure 7B). The latter model is supported by: i) significant enrichment of the GABPA CBRs in proximal promoters of convergent SAGPs from the 73-SAGPs set (Figure 8) and ii) the gene expression suppression effect in both gene partners of convergent SAGPs after siRNA *GABPA* knockdown in MCF-7 BC cells (Supplementary file 3: Figure S10).

**Figure 9:**
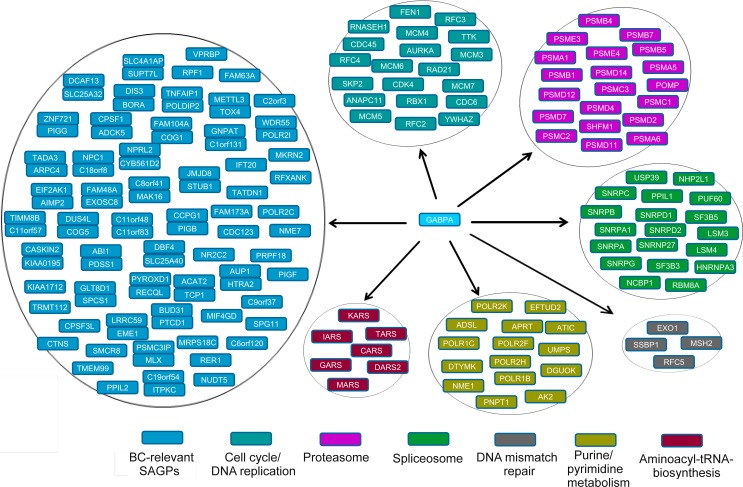
Pathological “core” gene set of GABPA gene network in BC Stacked pairs of genes indicate 73-SAGP gene partners which either share common GABPA CBR in their bidirectional promoter (divergent pairs) or have overlapping GABPA CBRs with proximal promoters of both gene partners (convergent pairs).

Stratification of BC patients using the SAGS followed by DEG and FA/GO analyses allowed us to extend the GABPA-dependent gene transcriptional network. Similarly, the proximal promoters of DEGs up-regulated in HR patient subgroups (identified by the SAGS, the “KEGG genes”) were strongly enriched by TFBMs and CBRs for GABPA. However, the full GABPA gene network might be much wider, as indicated by the higher frequencies of co-localized CBRs for *GABPA* in NGNs/PNGs than in the total genome gene set (Figure 8B). Therefore, the 73-SAGPs combined with the KEGG genes could be the representative “core” gene set of the GABPA gene network (Figure 9). The identified GABPA CBRs overlaps with the proximal promoters of *SKP2* and *AURKA* are in concordance with literature [54, 55].

We speculate that the 73-SAGPs within the GABPA gene network have a specific functional impact in BC progression and clinical outcome. Firstly, GABPA-dependent genes are involved in the regulation of cell entrance into the S phase of the cell cycle, independent of E2F1 [48]. Secondly, tight coordination and rewiring of the expression between their gene partners via the other SA related mechanisms [4]) could provide additional advantages for BC cells.

The discovery of specific breast tumor subgroups using SAGS and the GABPA gene network was possible due to our original, biological knowledge-driven, genomic architecture-centered meta-analysis approach (Figure 1). We highlight the importance of the preliminary pre-selection of SAGPs using correlation analysis, followed by the application of the 2-D RDDg developed for optimal survival analysis of SAGPs (Supplementary file 3: Figure S7). In the framework of this study, we assumed that genes that are evolutionary organised into SAGPs could acquire additional structural features and functions, providing certain advantages not only for the development of normal tissues but also in the tumorigenic process for certain subsets of BC tumors and can be regulated via distinct molecular factors/mechanisms.

Despite the extreme complexity of the human genome, the selection of appropriate negative controls (NGNs and PNGs, Supplementary file 2: Methods and Analyses) for the studied object (BC-relevant SAGPs) made it possible to identify and partially characterize the phenomena of the BC-relevant SAGPs as well as their potential mechanistic regulators.

For the first time, we found that cell cycle, proteasomal and spliceosomal gene sub-networks can be co-activated via GABPA in the same high risk BC patients (Figure 9), which might be useful for future clinical studies and practice in BC. Bortezomib, as an anti-proteasome agent (targeting the 20S-proteasome subunit) is an FDA-approved drug for multiple myeloma and is actively involved in several phase I/II BC clinical trials, including in combination with standard chemo- and endocrine therapies [56-58]. Anti-spliceosome drugs, as a novel treatment for cancer have been actively discussed in the literature (Supplementary file 2: Methods and Analyses), although they are currently just in the pre-clinical stage [59]. Recently, a novel drug targeting the 19S-proteasome subunit, b-AP15, was identified and successfully tested in a pre-clinical study against several cancers, including BC [60]. b-AP15-dependent targeting of proteasome [61] or siRNA-mediated targeting of spliceosome components [62] resulted in the same specific cellular phenotype: autophagy and reduction of viability in highly malignant BC cells. Hence, in the context of future clinical trials and the GABPA gene network (Figure 9), it is possible to suggest some alternative options for clinical treatments to improve BC patient outcomes: i) traditional chemotherapy combined with anti-spliceosome treatment and ii) anti-proteasome therapy combined with anti-spliceosome treatment.

The identified set of concordantly co-regulated 73-SAGPs represents a potential gene pool for further studies of the regulatory mechanisms of known or promising novel gene candidates involved in BC tumorigenesis and tumor progression.

Figure 8D shows an example of the convergent pair *DIS3/BORA* as one of the proposed 73-SAGP candidates for a future study of G3 basal-like breast cancers and cell cycle regulation in BC. *DIS3,* encoding the exosome endoribonuclease and 3′-5′ exoribonuclease, is a highly conserved gene required for mitotic progression and is involved in several cancers [63]. Silencing of *DIS3* alone affects the viability, migration and invasion of cancer cells [63]. AURKA and PLK1 are direct interactors of BORA at the G2/M transition in the cell cycle. The search of anti-cancer drugs targeting these genes to modulate mitosis is actively ongoing [64], but the results are controversial [65]. Firstly, the *DIS3/BORA* SAGP is significant and synergistic in terms of patients survival in two independent BC patients cohorts (Figure 4 and Supplementary file 1: Table S8); both gene partners are significantly correlated and activated in basal-like breast tumors (Supplementary file 3: Figure S1C and Supplementary file 3: Figure S1C). Secondly, GABPA is their common regulator in BC cells (Figure 8D and Supplementary file 3: Figure S10). Thirdly, in highly malignant HeLa cells, the cell cycle time-course expression of both genes is significantly associated with cell cycle periodicity (p(per) = 0.009 and p(per) = 5.1E-15 for *DIS3* and *BORA*, respectively) and is mutually coordinated in cell cycle phases (Supplementary file 3: Figure S9) [66].

Another candidate to study translation and oncogenesis is the convergent *AIMP2/EIF2AK1* SAGP (Table 2). AIMP2(p38) is a crucial component of the macromolecular aminoacyl-tRNA synthetase. The full size AIMP2 isoform has tumor suppressive properties based on the protective interaction with p53. In contrast, the alternatively spliced isoform AIMP2-DX2 is oncogenic and compromises the pro-apoptotic activity of normal AIMP2 through competitive binding to p53 [67]. Fusion gene *EIF2AK1-ATR* is oncogenic and overexpressed in androgen-independent prostate cancer cells [68]. *EIF2AK1*, encoding *the* translation elongation factor kinase, and *AIMP2* are involved in regulation of translation. Both genes were positively correlated in basal-like G3 tumors in 3 independent BC cohorts (Supplementary file 1: Table S1B) and are involved in the pathological GABPA gene network. Several other deregulated components of the same aminoacyl-tRNA synthetase complex were identified in the GABPA gene network (Figure 9).

The 73-SAGPs might also be investigated in context of locus-specific antisense modulation of known or novel oncogenes [20, 21]. In this scenario, complete direct blocking of a targeted abnormally activated sense gene (e.g. oncogene) can lead to undesirable side effects; however, experimental perturbation of its concordantly co-activated antisense partner could optimize the expression level of its deregulated oncogenic sense partner. This “soft modulation” model was [20] based on the previous detailed experimental studies of individual SAGPs, such as *TP53/WRAP53* [8] in various cancers and *BACE1/BACE1-AS* in Alzheimer disease [7].

**Figure 10:**
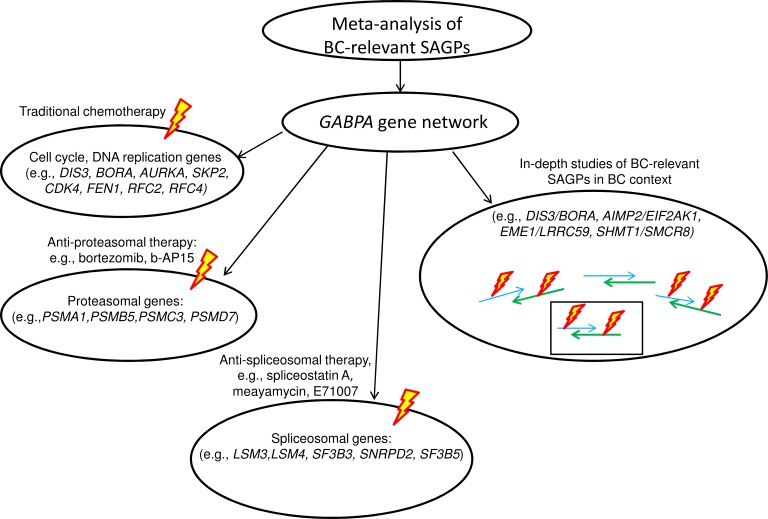
Meta-analysis of BC-relevant SAGPs and potential applications

The meta-analysis approach and the proposed data-driven model of the abnormally activated GABPA gene network in BC could be used in potential applications (Figure 10). Our model proposes: i) potential drug targets for anti-proteasome and anti-spliceosome therapy within the same GABPA gene network, in addition to traditional adjuvant chemo- and hormonal treatment and ii) the 73-SAGPs representing a pool of co-expressed paired genes could be used for in-depth studies of fine regulatory mechanisms of tumorigenesis and tumor progression in BC (Supplementary file 1: Table S13 and Figure 9). The latter option also looks promising in case of a progress of RNA-based drugs development and delivery in the nearest future [69, 70].

Thus, we used our integrative approach to identify novel aspects of the coordinated pathological gene networks in cancers. This study provides novel promising hereditary linked gene pairs associated with BC pathology as well as new facts and knowledge for further in-depth mechanistic studies. Our results suggest that SAGPs as highly-specific and important components of genomic organization in normal cells and pathological conditions can be useful for the search for alternative therapeutic targets.

## MATERIALS AND METHODS

### Breast tumors, normal breast tissue samples and microarray datasets

The collection of published datasets and our BC dataset used in this study are summarised in Supplementary file 2: Table S11.

The first dataset consisted of samples from the Uppsala cohort, which represented BC patients resected in Uppsala County, and the Stockholm cohort, which was derived from BC patients operated on at the Karolinska Hospital [27]. The second dataset consisted of samples from 251 BC patients from France collected at the Institute Paoli-Calmettes and Hospital Nord (Marseille) [71]. The Harvard cohorts 1 and 2 datasets included primary breast tumors obtained as anonymous samples from the NCI-Harvard Breast SPORE blood and tissue repository [72], [73]. The dataset of BC patients sample from the John Radcliffe Hospital, Oxford, UK and Guy's Hospital, UK, were selected from a multicentre study [74].

To obtain the additional training and testing groups used to verify the SAGS, we combined the microarray expression datasets from 5 independent BC cohorts (Metadata: combined the Oxford, the Guys hospital (GSE6532, GSE9195), the Harvard 2 (GSE19615), the Marseille (GSE21653) and the BII-OriGene cohorts (GSE61304), (Table 1 and Supplementary file 1:Table S12) with a consequent batch effect correction using the ComBat software [75]. The quality of the combined datasets was monitored using the arrayQualityMetrics software [76].

The normal breast tissue microarray data included normal epithelium tissue samples resected from benign non-malignant lesions of women with a non-elevated risk of BC undergoing reduction mammoplasty (fist dataset [77], RM, *n* = 18). The second [77] (HN, *n* = 18) and third [78] (AH, *n* = 62) datasets consisted of histologically normal “tumor-adjacent” (i.e., located from 1cm to 2 cm from the tumor) epithelium samples obtained from groups of women undergoing BC surgery who had not undergone chemotherapy or radiation treatment before tissue acquisition. The first and second datasets were age-matched. For these datasets, the 53 SAGPs out of the total set of 73-SAGPs, in which both members of a gene pair were supported by at least 1 Affymetrix U133A probe sets, were used for correlation analysis; all breast tissue samples were obtained by laser-capture micro-dissection [77]. In the third dataset, data on both U133A and U133B microarrays were available, so all 73-SAGPs and 73 pairs of NGNs were used for the correlation analysis.

### Survival prediction analysis

The Cox hazards model was used to parameterize and compute the differences between the Kaplan-Meier survival curves. *P*-values of the Wald test statistics were used to evaluate the statistical differences between the survival curves.

The 1-D DDg approach was used for the selection of individual genes where expression threshold (cut-off) value could be used to group the patients into distinct disease development risks according to the survival time-to-event data [29]. Briefly, the patients were sorted-out according to the expression values of a tested gene and the gene expression values were fitted to survival times and corresponding events (e.g., disease free survival, DFS) using the Cox proportional hazards model; the optimal gene expression cut-off value for each gene was estimated by goodness-of-fit analysis on *a one-dimensional linear scale*, maximising the separation between the sorted-out patients into low- and high-risk subgroups, represented by Kaplan-Meier survival curves [29].

Such survival prognostic methods utilise expression data for individual genes as the features for survival prognosis [34, 79]. In the cases of gene pairs, the method may be improved and/or specified by analysing gene pairs. Specialised statistical and computational methods are required to reliably identify gene pairs (e.g., the predictive interaction analysis (PIA) [79, 80] or the 2-dimensional data-driven grouping procedure (2-D DDg) [29, 34]). Due to the sample size, cohort variation and the computerized implementation of a mathematical model, the selection of unbiased and high-confidence gene pairs was not a trivial task. The 2-D DDg method [29, 34], which is based on a non-linear, unsupervised prognostic and feature selection model, can accurately classify the most common patterns (designs) of gene relationships in pairs and explicitly include interaction (synergy) effects in its statistical procedure.

In the current study, gene pairs-based survival prediction analysis was performed using either the previously developed 2-D DDg [29, 34] and/or its substantially improved extension, the 2-D RDDg (Figure 3). In the 2-D DDg, in contrast to the 1-D DDg, dichotomization of patients into distinct risk subgroups was performed for each gene pair on a *2- dimensional plane* with horizontal and vertical axes corresponding to the fixed gene expression value cut-offs. Our 2-D DDg method is also distinct from the known PIA approach [79] because i) it is described by a 2- dimensional (for two interacting genes), not a linear, statistical model (when just one value for a two-gene ratio or a two-gene product is used); ii) the 2-D statistical model is more informative than the gene ratio-based model for patients survival partition because it uses the same 2 genes, 5 designs and 10 sub-designs (i.e., 10 prognostic scenarios, Supplementary file 3: Figure S6) in contrast to only 2 possible scenarios for the same gene pair in the ratio-based model.

Because both genes of an SAGP are often significantly correlated between each other (Figures 2 and 4), the bi-variate distributions of their gene-partner expression values could deviate from a random “shotgun” shape (Figure 4A1 and 4A2). In such cases, the 2-D DDg model coordinates might be not optimal to reveal the best survival patient partition in the 2-D gene expression space. In the 2-D RDDg analysis (Figure 1), in contrast to the 2-D DDg (Supplementary file 3: Figure S6), the horizontal and vertical axes can be rotated at a varied angle without losing their orthogonality. The 2D-RDDg utilizes 7 designs, 14 sub-designs and 16 rotation angles. The rotation property allows the 2D-RDDg to be more flexible and to refine more accurate patient partitions than can be achieved using the 2-D DDg.

The WVG procedure was used to combine the survival information for multiple gene pairs into an essentially improved integrated grouping (Supplementary file 2: Methods and Analyses). Individual classification patterns for selected survival significant genes or gene pairs are organised in a matrix in a fixed order. Voting procedure in the matrix is performed step-by-step for each gene pair in descending order; for each individual patient, the predominant number of votes for each class (”0” or ”1”) from all the genes/gene pairs in a given list is used for the final integrated assignment of the patient to a corresponding class (the low-risk “0” or the high-risk “1” classes).

To stratify patients in diverse BC cohorts, we used the refined 2-D RDDg procedure due to its higher accuracy (Supplementary file 3: Figure S7, Supplementary file 2: Methods and Analyses). However, the 2-D DDg procedure is much faster to execute and, therefore, it could be useful for massive screening purposes.

### Cross-cohort and cross-platform reproducibility of SAGS

For cross-cohort validation of the SAGS (Table 2), patient stratification was considered significant if two identified novel patients subgroups (high-risk, HR and low-risk, LR) showed differences in the WVG Wald test with *p*-value < 0.01. Patient stratification in the training data set was considered valid if the significantly different novel survival subgroups were identified in a corresponding independent testing data set. The BC cohorts used in the training and testing modes are summarised in Table 1.

For qRT-PCR validation of the SAGS, we designed a strand-specific qRT-PCR protocol for 9 of the 12 SAGPs from SAGS (eighteen genes, Supplementary file 2: Table S10A) to exclude a potentially undesirable gene-expression signal from an opposite DNA strand within the SA overlap region. Forty-two breast tumors (RNA samples; OriGene Technologies, Rockville, MD) were stratified in parallel using either the U133Plus 2.0 microarray (Figure 5E) or qRT-PCR expression data (Figure 5F) for the same genes and patients. The 2-D RDDg and WVG procedures from the training mode were independently applied to both data sets.

### Microarray analysis of the BII-OriGene cohort

Total RNA, histopathological data, tumor sample images and clinical data from 58 BC patients were obtained from OriGene Technologies (Rockville, MD). Microarray analysis was performed according to the standard Affymetrix chip protocol (Supplementary file 2: Methods and Analyses).

### Strand-specific quantitative RT-PCR

cDNA was synthesised from the total RNA (250 ng) of 42 BC patient samples purchased from OriGene Technologies (Rockville, MD) using a gene-specific pool of reverse primers specific for the sense/anti-sense transcript regions. Oligoprimers were designed to fall within specific regions of the corresponding Affymetrix probe sets. The SA cDNAs of 42 patient samples were pre-amplified (Life Technologies, Taqman PreAmp Master Mix kit) using a gene-specific pool of SA forward and reverse primers. TATA box binding protein (TBP) was used as endogenous control. Taqman probes were designed for all the sense and anti-sense genes, as well as the endogenous controls. The 96 × 96 Dynamic Array IFC was prepared according to the manufacturer's instructions (Fluidigm, San Francisco, CA), as described previously [81]. Quantitative PCR was performed using a gene assay (1^st^ BASE, Singapore), according to the protocol of the Biomark System (Fluidigm, San Francisco, CA). Reaction conditions were as follows: 50°C for 2 min, 70°C for 30 min, 25°C for 10 min, 50°C for 2 min and 95°C for 10 min, followed by 40 cycles of 95°C for 15 sec and 60°C for 60 sec. Ct values were extracted, and the data were processed using detector thresholds individually set for each gene and a linear baseline correction using the Biomark Real-time PCR Analysis software (v.3.0.4) (Fluidigm, San Francisco, CA). The genes were relatively quantified using the dCt method [82]. A list of forward and reverse primers for both sense- and anti-sense genes and the respective fluorescent Taqman probes labelled with a FAM-TAMRA quencher is provided in Supplementary file 2: Table S10A.

### siRNA knockdown assay

MCF-7 cells were cultured in EMEM supplemented with 10% FBS in a humidified incubator at 37°C with 5% CO_2_. For *GABPA* knockdown experiment MCF-7 cells were transfected with ON-TARGETplus siRNA duplexes targeting *GABPA* mRNA (Dharmacon) and negative non-targeting control RNA (siGenome non-targeting RNA, Dharmacon) using Dharmafect1 reagent according to manufacturer's instructions. Cells were harvested 72 hours after transfection and total RNA was extracted using RNeasy Mini Kit (Qiagen) according to manufacturer's instructions. We assessed gene expression after *GABPA* knockdown in 22 genes from 11 convergent SAGPs and in 12 spliceosomal and proteasomal genes. To minimize chance of cross-contamination from opposite DNA strand in the SAGPs, we designed primers pairs for conventional qRT-PCR outside of the regions of SA overlaps, predominantly within the first half of a gene (5′-end) (Supplementary file 2: Table S10B). Total RNA was used as a template for reverse transcription using QuantiTect Reverse Transcription Kit (Qiagen) using random hexamer primers. The transcripts were analyzed by qRT-PCR run on a Quant Studio 6 Flex System (Applied Biosystems). The genes were relatively quantified using the dCt method [82].

### DNA copy number variation analysis

We estimated CNV for each gene from total sets of 146 genes of 73-SAGPs and 146 NGNs in two independent BC datasets. The first BC dataset included 38 BC cell lines for which both CNV and gene expression microarray data were available [32]; the second dataset comprised CNV data for 93 primary breast tumors [83].

Assignment SNPs to genes was performed using the Galaxy platform [84] by first joining SNPs to the gene intervals followed by fetching additional closest SNPs located upstream and downstream to the gene. DNA copy number value for each gene was estimated as an average of CNV values for all SNPs assigned for the gene (Supplementary file 1: Tables S4A and S4B). The analysis revealed that 21 SAGPs in the first dataset and 5 SAGPs in the second dataset were located in moderately or highly amplified regions of the genome.

### Transcription factor binding motifs and CBR analyses in the proximal promoters of 73-SAGPs and other gene sets

We analysed the proximal promoters (−450/+50 bp) for the enrichment of transcription factor binding motifs using PSCAN software [42] in the following sets of genes: i) 146 genes of 73-SAGPs; ii) 147 genes identified by SAGS as significantly over-expressed in HR BC subgroups, significantly over-represented under the category “KEGG pathway” and related to the proteasome, cell cycle, DNA replication, spliceosome, aminoacyl-tRNA biosynthesis and purine/pyrimidine metabolism (Supplementary file1: Tables S9B, S9C and S13, the “KEGG genes” set). The following 3 independent negative control sets were used: i) the set of 102 genes of PNGs (Supplementary file 3: Figure S4, Supplementary file 2: Methods and Analyses), ii) the set of 146 NGNs and iii) the set of 150 DEDR genes (Supplementary file 1: Table S9C).

To verify our *in-silico* predictions, we utilised the publicly available ChIP-seq data for GABPA (MCF-7 breast cancer cells, GEO ID: GSM1010864) generated by ENCODE (www.genome.ucsc.edu/ENCODE/). The ERα ChIP-seq data for MCF-7 cells were downloaded from GEO ID: GSE48930 [85]. For each studied gene set, we first identified higher confidence CBRs (reproducible group) via the identification of common overlapping significant peaks (see descriptions in GSE31477 for *GABPA*) between all available ChIP-seq replicates (2 replicates for GABPA and 3 - for ERα). Then, for each set of higher confidence CBRs taken from the first replicate for each transcription factor, we identified the overlapping regions of the CBRs with the proximal promoters (+50/−450 bp) in each studied gene set. Genomic interval manipulations were performed using the Galaxy platform [84].

Cytoscape (version 3.2.1) was used for visualization of GABPA gene network [86].

### Accession Numbers

The microarray data for BII-OriGene BC cohort are deposited at the GEO database (http://www.ncbi.nlm.nih.gov/projects/geo/) under the accession ID (GSE61304).

### Ethics statement

An ethics statement was not required for this work.

## SUPPLEMENTARY MATERIAL FIGURES AND TABLES







## Abbreviations

BC: breast cancer
SA: sense-antisense
SAGP: sense-antisense gene pair
SAGS: sense-antisense gene signature
73-SAGPs: 73 BC-relevant SAGPs
2-D RDDg: 2-Dimentional Rotated Data-Driven Grouping
WVG: Weighted Voting Grouping
NGN: nearest gene-neighbor
PNG: pair of reproducibly correlated non-overlapping neighboring genes
LR: low risk
HR: high risk
KS: Kolmogorov-Smirnov test
WMP: Wilcoxon matched pairs test
ROC: receiver operating characteristics
AUC: area under the curve
FA/GO analysis: Functional Annotation and Gene Ontology analysis
DEG: differentially expressed genes
snRNPs: small nuclear ribonucleic particles
CBR: ChIP-seq binding region
ChIP-seq: chromatin immunoprecipitation sequencing
ERα: estrogen receptor alpha
