# Supplementary material for: Sense-antisense gene-pairs in breast cancer and associated pathological pathways
Source: Oncotarget. 2015 Oct 28;6(39):42197–221. doi: 10.18632/oncotarget.6255 (PMC4747219; doi:10.18632/oncotarget.6255)
Supplement: Supplementary file 1 [file oncotarget-06-42197-s001.pdf]

Supplementary Information

Sense-Antisense Gene-Pairs in Breast Cancer and Associated Pathological Pathways

Oleg Grinchuk, Efthymios Motakis, Surya Pavan Yenamandra, Piroon Jenjaroenpun, Ghim Siong Ow, Zhiqun Tang, Aliaksandr Yarmishyn, Anna V. Ivshina, Vladimir A. Kuznetsov

Supplementary Table S2. Comparison of Kendall's Tau correlation coefficients distributions between sense and antisense gene-partners for the set of the 53-SAGPs (Supported by Affymetrix U133A array signals).

	HN	RM	BlG3(U)	nBlG3(U)	BlG3(S)	nBlG3(S)
HN		*D = 0.26, p =0.082*	**D = 0.38, p =1.1e-03**	**D = 0.42, p =2.2e-04**	**D = 0.40, p =4.9e-04**	**D = 0.45, p = 3.8e-05**
RM			**D = 0.26, p = 0.049**	**D = 0.30, p = 0.016**	D = 0.23, p = 0.132	**D = 0.28, p = 0.028**
BlG3(U)				*D = 0.15, p = 0.582*	D = 0.13, p = 0.7444	D = 0.17, p = 0.4294
nBlG3(U)					D = 0.15, p = 0.581	D = 0.11, p = 0.886
BlG3(S)						*D = 0.15, p = 0.582*

D-values and p-values were calculated using Kolmogorov-Smirnov test for each pair of samples. Bold font indicates significantly different pairs of distributions ($p<0.05$). Italics underlined font indicates that differences between basal-like G3 subtype and non-basal-like subtype in each of three breast cancer cohort as well as between two normal tissue controls were non-significant.

HN - histologically normal epithelium samples from breast cancer patients (n=18) [1]; RM - cancer free reduction mammoplasty samples from healthy individuals (n=18) [1]; BlG3(U) – basal-like breast tumor samples (Grade 3) from Uppsala cohort (n=24) [2]; nBlG3(U)– non-basal-like breast tumor samples (Grade 3) from Uppsala cohort (n=31) [2]; BlG3(S) – basal-like breast tumor samples (Grade 3) from Stockholm cohort (n=18) [2, 3]; nBlG3(S) –non-basal-like breast tumor samples (Grade 3) from Stockholm cohort (n=28) [2, 3].

Supplementary Table S6. Literature analysis of the survival significant genes in the Uppsala and Stockholm cohorts.

RefSeq gene symbol	Gene description	Association with cancer(s)	Reference
ADCK5	aarF domain containing kinase 5	-	
ARRDC1	arrestin domain containing 1	-	
BCCIP	BRCA2 and CDKN1A-interacting protein	Important cofactor for BRCA2 in tumor suppression; Modulator of CDK2 kinase activity via p21; prognostic marker for radiotherapy of laryngeal cancer	[4],[5]
BOLA2	bolA homolog 2 (E. coli)	-	
C11orf48	chromosome 11 open reading frame 48	Potential tumor antigen marker in bladder cancer	[6]
C11orf57	chromosome 11 open reading frame 57	Gene is deleted in a patient of head and neck paraganglioma	[7]
C2orf3	chromosome 2 open reading frame 3	Association with childhood acute lymphoblastic leukemia	[8]
C6orf120	chromosome 6 open reading frame 120		
CCNE2	cyclin E2	Prognostic marker for lymph node-negative breast cancers	[9]
CCPG1	cell cycle progression 1 (CCPG1)	Alteration in cell cycle progression	[10]
CYB561D2	cytochrome b-561 domain containing 2	Tumor suppressor in lung cancer, potential drug target	[11]
DBF4	activator of S phase kinase	Activating subunit of CDC7. Together with CDC7 demonstrates overexpression upon p53 loss in various tumors.	[12],[13]
DCAF13	WD repeats and SOF1 domain containing	-	
EME1	essential meiotic endonuclease 1 homolog 1	Potential sensitivity marker for cisplatin-based chemotherapy in various tumors	[14]
KMO	kynurenine 3-monooxygenase	-	
LRRC59	leucine rich repeat containing 59	-	
MIF4GD	MIF4G domain containing	-	
MRPL19	mitochondrial ribosomal protein L19	-	
MRPS25	mitochondrial ribosomal protein S25	-	
NUDT5	nudix-type motif 5	-	
POLR2I	DNA directed RNA polymerase II polypeptide I	-	
RHOQ	ras-like protein TC10	-	
STUB1	STIP1 homology and U-box containing protein 1	Tumor suppressor in breast cancer, inhibits metastatic potential by degrading oncogenic proteins including SRC-3 and ERalpha	[15],[16]
TATDN1	TatD DNase domain	-	

	containing 1		
TCP1	T-complex protein 1	Inhibition of TCP1 is associated with enhanced cisplatin sensitivity in ovarian cancer	[17]
TMEM97	transmembrane protein 97	Expression of TMEM97 is associated with survival in metastatic colorectal cancers	[18]
TOX4	epidermal Langerhans cell protein LCP1	Involved in cell response generated by platinum anticancer drugs	[19]

Supplementary Table S7. Literature analysis of the genes comprising twelve synergistic survival significant SAGPs (SAGS).

RefSeq gene symbol	Gene description	Associations with cancer(s)	Reference	RefSeq gene symbol	Gene description	Association with cancer (s)	Reference
C18orf8	chromosome 18 open reading frame 8			NPC1	Niemann-Pick disease, type C1 precursor	NPC1 activity is associated with the emergence of multidrug resistance of HL-60 cancer cell line	[20]
C13orf34	aurora borealis (BORA)	Radiation sensitivity in cancer; breast cancer; activator of the protein kinase Aurora A; control of mitosis	[21-23]	DIS3	DIS3 mitotic control homolog (S. cerevisiae)	Differentially expressed in colorectal carcinoma; control of mitosis	[24, 25]
AIMP2	Aminoacyl tRNA synthetase complex-interacting multifunctional protein 2	Tumor suppressor in lung and ovarian cancer	[26, 27]	EIF2AK1	Eukaryotic translation initiation factor 2-alpha kinase 1	Fusion gene EIF2AK1-ATR was predicted to be oncogenic in androgen-independent prostate cancer cells	[28]
SHMT1	serine hydroxymethyl-transferase 1	Associations with rectal and intestinal cancers	[29]	SMCR8	Smith-Magenis syndrome chromosome region, candidate 8		
DOK4	docking protein 4	Altered expression in clear cell renal cell carcinoma	[30]	POLR2C	DNA directed RNA polymerase II polypeptide C	the POLR2C rs4937 polymorphism is associated with the response to the oxaliplatin-based chemotherapy	[31]
MRPS18C	Mitochondrial ribosomal protein S18C			FAM175A	Family with sequence similarity 175	BRCA1-A complex subunit; potential marker of platinum-gemcitabine chemotherapy	[32]
CTNS	Cystinosin, lysosomal cystine transporter			TAX1BP3	Tax1 (human T-cell leukemia virus type I) binding protein 3	Oncogenic protein, potential marker of invasive breast tumors	[33]
EME1	Essential meiotic endonuclease 1 homolog 1	Potential sensitivity marker for cisplatin-based chemotherapy in various tumors	[14]	LRRC59	Leucine rich repeat containing 59	Proteomic study revealed that LRRC59 is strongly associated with aggressiveness of metastasis in BC	[34]

VPRBP	Vpr (HIV-1) binding protein	Inhibitor of p53 target genes	[35]	RBM15B	RNA binding motif protein 15B		
RNF139	Ring finger protein 139	Putative tumor suppressor in kidney cancer	[36]	TATDN1	TatD DNase domain containing 1	Fusion gene in breast cancer	[37]
SSB	Sjogren syndrome antigen B			METTL5	Methyltransferase like 5		
BIVM	basic, immunoglobulin-like variable motif			KDELC1	KDEL (Lys-Asp-Glu-Leu) containing 1		

Supplementary Table S10A. Oligoprimers and TaqMan probes used for strand-specific qRT-PCR in nine SAGPs (eighteen genes) and two internal controls.

Host Gene Symbol and transcript ID, corresponding to the Affymetrix probeset	Strand	Affymetrix probeset	Sequences of oligoprimers used in QRT-PCR		TaqMan probe
			Forward primer	Reverse primer	
BORA	+	A.219544_at	5'-CTTTTCAATGCAGCAGTCCA-3'	5'-TTCCAGGAGCATTTCTGTTG-3'	FAM-TGCCTCTGTCAGAATCAAAGACTAAGC-TAMRA
DIS3	-	A.218362_s_at	5'-ATGTTACCATGGCTGTGTCC-3'	5'-ACCTAGCCGGCAAACTTTTT-3'	FAM-CCACCCCCGCCAACCATTT-TAMRA
AIMP2	+	A.209971_x_at	5'-GGGAACATTGCACGTTTCTT-3'	5'-AGAGTTCATGGAGCGGAAAA-3'	FAM-TGCTGTCAACGCAACCCTTA-TAMRA
EIF2AK1	-	A.217736_s_at	5'-GCCTGCACATACTCACTGGA-3'	5'-CTTTGCAAGGGTGTGGTTTT-3'	FAM-CCAGTCTTCCTGCCCCTTTC-TAMRA
POLR2C	+	A.208996_s_at	5'-GTTGAGCTTCTTGGCAGGAC-3'	5'-AGGTGCACTGGAAACACTCC-3'	FAM-ACCAGGGATGCAGTGGTGTT-TAMRA
DOK4	-	A.209690_s_at	5'-CAAGGGCACGGAACATTACT-3'	5'-GCTTTGGCTTTAGCAGGATG-3'	FAM-CAGTGCCTACTGGCACCACA-TAMRA
MRPS18C	+	B.228019_s_at	5'-ACTTGGTAACGGCTGCTGTC-3'	5'-TGCCTTCCATAAATGCATCC-3'	FAM-CTCACACGGTGCTTTGGAGAA-TAMRA
FAM175A	-	B.226521_s_at	5'-TGAGCTATTTGGGGATCAGA-3'	5'-TGACTCAAACCAACCTTTGGA-3'	FAM-TGGACATTGGTATGAAGGGGA-TAMRA
CTNS	+	A.204925_at	5'-AGATTAGCCCCATCTGAGCA-3'	5'-CAGGCCAGAAATTGGTGAGT-3'	FAM-CCCAGCATCTGGAGTACAGGA-TAMRA
TAX1BP3	-	A.209154_at	5'-CACCTATCAGGGCAGCTCTC-3'	5'-TAAGGCTTGTGCACTTGCTG-3'	FAM-TTCCTCTTTGCCTGTGGGTTTA-TAMRA
EME1	+	B.234464_s_at	5'-TTGAAGTCGTGAGTGAAGCTG-3'	5'-TTGGTACAAATGCTTTATTGAAAC-3'	FAM-CTTGACTGGGCTCAGCCACTGA-TAMRA
LRRC59	-	B.234812_at	5'-GGCCAAAAAATTTGCTTTCCT-3'	5'-TGCAGGCTGATATCATAGGC-3'	FAM-TGCTTATTACATTGTGATCTGACCTG-TAMRA
RBM15B	+	A.202689_at	5'-AGGACATTGGGCAAGCTAGA-3'	5'-GTTTTGGTGGTGGACAGGAC-3'	FAM-CCTGTCTTTCCCAGCGTCAT-TAMRA
VPRBP	-	B.226481_at	5'-TGCCTCATATGCTGTCAGGT-3'	5'-TTTCCACAATGGGGAGAAAG-3'	FAM-TTTCCTTCGTGCCAGAGGTG-TAMRA
SSB	+	A.201139_s_at	5'-GTGCAACTGGACCTGTGAAA-3'	5'-CAGGTGGACATTGAAGTGGA-3'	FAM-ACTTTTGTTTGCGGGGCTTT-TAMRA
METTL5	-	A.221570_s_at	5'-GCATCGGAACTGCAATGTTA-3'	5'-GTCCCAAAGGGAGGATTCAT-3'	FAM-CATGGTTCAATGTGATGTGTGC-TAMRA
BIVM	+	B.222761_at	5'-TTTTCTTCTGCTGCCAACCT-3'	5'-CCTTTTCCTTGAGGTGTATTGC-3'	FAM-GGGTCTTGTTCCAGGACTCAAA-TAMRA
KDELC1	-	A.219479_at	5'-GTGCTGAAGCAGGATTCCAT-3'	5'-TGTCATCGCCCATGAGATTA-3'	FAM-CTGCAGCCCTGGAAACACT-TAMRA
ACTB	-	NA	CAGCCATGTACGTTGCTATCCAGG	AGGTCCAGACGCAGGATGGCATG	FAM-ACTGGCATCGTGATGGACTC-TAMRA
TBP	+	NA	TGCCCGAAACGCCGAATATAATC	GTCTGGACTGTTCTTCACTCTTGG	FAM-CAAGCGGTTTGCTGCGGTAATC-TAMRA

Supplementary Table S10B. Oligoprimers used for conventional qRT-PCR after *GABPA* knockdown in MCF-7 cells.

Host Gene Symbol	# of the SAGP	Sequences of oligoprimers used in QRT-PCR	
		Forward primer	Reverse primer
BORA	1	5'- AGCTCCCTGGAGTCGGTACT -3'	5'- AAACAGAAGGACTGGCGAGA -3'
DIS3		5'- AACTGCTAACCCCGAACTCA -3'	5'- TTGTCGCCATGAATCCATAC -3'
PYROXD1	2	5'- ATCCGTGATACAGACAGTGCT -3'	5'- GCAATACCACCGTTCCCTATGAT -3'
RECQL		5'- CGGCAGTTCCCTAACGCAT -3'	5'- TCTTCAGTGTTTGAGGGCTTCT -3'
ABI1	3	5'- ACCAGTCCTGCTAGGCTTG -3'	5'- ACTGTTTTCTCGACTTCCACTTC -3'
PDSS1		5'- CGCCATAGCCTTAATTGCAGA -3'	5'- GAACTTGCATCGTCAATAACGTC -3'
BUD31	4	5'- GCTGGGAGTTGATTGAGCCAA -3'	5'- CCACAGAGATTCCACTTTCCTC-3'
PTCD1		5'- CCTCTCTGACAAATACTCCTCCC -3'	5'- CCAAACCGCAGGTTATGGAAC -3'
EXOSC8	5	5'- CGGTTCAATTAGTACCGCAGAT -3'	5'- ACGTATCCTTTATCAGGGGCAT -3'
FAM48A		5'- TTGCGGATGTGTCATAGCAGA -3'	5'- AATGTGCCGACTTTGGTAACC -3'
ITPKC	6	5'- TGGACACAACCTAGCACTGAC -3'	5'- TGGTTCCTCTAATGGGCCATC -3'
C19ORF54		5'- CCACGGAAAGAGGCTACACG -3'	5'- CAGGGGATGTCCAGTGACC -3'
ACAT2	7	5'- CCCAGCCAATGCTTCAGGAAT -3'	5'- AAGCCCACGTTTATCAGCTTC -3'
TCP1		5'- CCAGCCACGCTATCCAGTC -3'	5'- TCAAGGCAAGCAATTTTTGCAT-3'
COG1	8	5'- CTGCCCAGATCAAGCTACTCT -3'	5'- GGGACTGTATCGGGAACTAGAAG -3'
FAM104A		5'- CCGGAAGTAGTGGTGGTACG -3'	5'- CTCCGCGAAGAGAGGGAAC -3'
MLX	9	5'- TGTCCACGTTACGCAAGGATG -3'	5'- GGTGTGCCTTCACAATCTGCT -3'
PSMC3IP		5'- GTGTTCGGGAACCTACAGCG -3'	5'- TGGTCCTGATCCGCAAAATAGA -3'
PIGB	10	5'- GTTCCAGATGAATACTGGCAGTC -3'	5'- GCAACTGAACACTATCTTTCCCT -3'
CCPG		5'- AGTGATGAAACCAGTAATCAGCC-3'	5'- GCAACTAGCCGGTCTTCAGAT-3'
ADCK5	11	5'- GTATCGGGGTGTCTACGGC -3'	5'- CCTGAGCGCCACTTCAAACT -3'
CPSF1		5'- CGCAGCTCTACGTGTACCG -3'	5'- GGACATGACGTTGCCAAAGAA -3'
SNRPC	NA	5'-ATGATGCCAGCACCCCATAT-3'	5'-GGGCAGGAGGTCTCATCATT-3'
LSM3	NA	5'-GGGAGATGTGGAAGAAACTGTG-3'	5'-TTCAGCCAACTCTCAGTGGA-3'
PPIL1	NA	5'-CAGCCAGTTCTTTGTGACCC-3'	5'-TGATCTTCACGTCGTCCACA-3'
SNRPD2	NA	5'-GGGCAAGAAGAAGTCCAAGC-3'	5'-CAGGACCACGATGACTGAGT-3'
SF3B5	NA	5'-ATGACTGACCGCTACACCAT-3'	5'-GTTGAGAAGGTCGAAGTGGC-3'
LSM4	NA	5'-GAAGTCATCTGCACGTCCAG-3'	5'-CACCTCCTCCTTGACCATGT-3'
PSMC2	NA	5'-AGAATCTGACACTGGCCTGG-3'	5'-CTGGCAACCTGTAAAGGCTG-3'
PSMC3	NA	5'-CCTGGTGGGTGTGAACAAAG-3'	5'-CTCCTGGATCTGCTTGTCCA-3'
PSMB4	NA	5'-GTCCACTCCCGATTCCTTCA-3'	5'-AACTTAACGCCGAGGACTGA-3'
POMP	NA	5'-TGCCAGAGGACTTGGATCTG-3'	5'-TCAAGGGGATGACTAGGCAA-3'
PSMB5	NA	5'-GTCCCAGAAGAGCCAGGAAT	5'-TCTGGGAGGCAATGTAAGCA-3'
PSMA1	NA	5'-CTCAATTGCGGGGCTTACTG	5'-TACAAGACGAGACACAGGCA-3'
SKP2	Positive control	5'- CCAGGAGATTCCAGACCTGA -3'	5'- CACTCCCTTTGCTCTTCAGC -3'
DCUN1D4	Negative control	5'- GCCGCCGCTGTCAATTTTC -3'	5'- AGGTTCAGCTTATTAAGGGTGTG -3'

Supplementary Table S11. Microarray datasets used in the present study.

Description of dataset	Type of microarray	Used samples, n	Series accession ID (GEO)	Ref.
Breast cancer tumors (Uppsala cohort)	Gene expression microarray, Affymetrix U133A&B	249	GSE4922	[2, 3]
Breast cancer tumors (Stockholm cohort)	Gene expression microarray, Affymetrix U133A&B	159	GSE1456	[2, 3]
Breast cancer tumors (Harvard cohort 1)	Gene expression microarray, Affymetrix U133 Plus 2.0	47	GSE3744	[38]
Breast cancer patients (Harvard cohort 2)	Gene expression microarray, Affymetrix U133 Plus 2.0	115 (103 in Metadata)	GSE19615	[39]
Breast cancer tumors (Marseille cohort)	Gene expression microarray, Affymetrix U133 Plus 2.0	250 (243 in Metadata)	GSE21653	[40]
Breast cancer tumors (Singapore cohort)	Gene expression microarray, Affymetrix U133A&B	88	GSE4922	[2, 3]
Breast cancer tumors (Oxford cohort +Guys hospital cohort), UK	Gene expression microarray, Affymetrix U133A&B	(68+116 in Metadata)	GSE6532	[41]
Breast cancer tumors (Guys Hospital cohort),UK	Gene expression microarray, Affymetrix U133 Plus 2.0	(58 in Metadata)	GSE9195	[42]
Histologically normal breast epithelium	Gene expression microarray, Affymetrix U133A	36	GSE20437	[1, 43]
Histologically normal breast epithelium	Gene expression microarray, Affymetrix U133 Plus 2.0	62	GSE10780	[44]
Breast invasive ductal carcinomas	Gene expression microarray, Affymetrix U133 Plus 2.0	30	GSE10780	[44]
51 breast cancer cell lines	Gene expression microarray, Affymetrix U133A', DNA CNV data	38	GSE12777	[45]
RNA samples of G1, G2 and G3 Nottingham grade breast tumors purchased from OriGene Technologies (Rockville, MD).	Gene expression microarray, Affymetrix U133 Plus 2.0	58 (57 in Metadata)	GSE61304	Current report

Supplementary Information

Sense-Antisense Gene-Pairs in Breast Cancer Progression and Associated Pathological Pathways

Oleg Grinchuk, Efthymios Motakis, Surya Pavan Yenamandra, Piroon Jenjaroenpun, Ghim Siong Ow, Zhiqun Tang, Aliaksandr Yarmishyn, Anna V. Ivshina, Vladimir A. Kuznetsov

Supplementary Methods and Analyses

Identification of BC-relevant SAGPs using Kendall's Tau correlation analysis

From the set of 728 SAGPs (see Results section in the main manuscript), we obtained 334 non-redundant SAGPs for which each gene-member was supported by at least one U133 A&B Affymetrix probeset (Table S1, A). Using microarray expression data from the Uppsala, Stockholm [2] and Harvard [38] cohorts, we selected two clinical subgroups in each cohort: a subset of grade 3 breast tumours, which belong to the basal-like subtype; and grade 3 breast tumours, which belong to the luminal A, luminal B, Normal-like and ERBB2 subtypes collectively termed the "non-basal-like" subgroup.

For each patient cohort and clinical subgroup, Kendall’s Tau correlation coefficients were calculated for the probeset pairs. High-confidence correlated probeset pairs ($p<0.05$) were selected, and we identified common probeset pairs (73 SAGPs, Table S1B and S1C) among the three cohorts and two subsets. Common SAGPs were selected via Gene Symbols. For genes with multiple probesets, we selected the probeset that displayed the highest correlation with its probeset partner for a given SAGP. Thus, we identified SAGPs that were meaningful for breast cancer as complex gene pairs architectures rather than as individual genes. We assumed SAGP as an overlapping transcriptional architecture[46] which is composed not only of protein coding-

coding SA host genes but includes all the transcripts within the territory of a SAGP and which could be tightly transcriptionally/post-transcriptionally related. Hence, we included also those Affymetrix probesets that might not represent a transcript with sense (or antisense) overlap, but still belongs to the same complex locus of a SAGP.

Negative control gene sets used for analyses of SAGPs.

The nearest genes neighbours (**NGNs**) set served as a negative control for SAGPs in correlation, DNA CNV and proximal promoters analyses, and it was selected using the following criteria:

1) the nearest upstream or downstream neighbouring gene for each gene partner of a SAGP together composing a nearest gene pair;

2) NGNs should not be involved in the same complex sense-antisense architecture [47] according to the RefSeq track of the UCSC Genomic browser;

3) every NGN should be a protein-encoding gene represented by a RefSeq NM_ID;

4) the NGN should not be the same for two different SAGPs, and it should be driven from the studied set of 73 SAGPs;

5) the NGN must be represented by at least 1 probeset from Affymetrix U133A&B platform (Tables S3A and S3B).

Additionally, we used the set of reproducibly correlated pairs of non-overlapping neighbouring genes (**PNGs**) identified using the same procedure and BC subgroups cohorts as 73-SAGPs for proximal promoter analysis; the procedure for their isolation is described in detail below.

We suggested a selection of the genes in the pairs located in vicinity of each other, which are located on opposite DNA strands, but not sharing common nucleotides (an essential property of sense-antisense gene pairs). According to this criteria, we found at least 2473 non-overlapping

neighbouring genes in total in the human genome (Figure S3). The total available genomic pool of non-overlapping neighbouring genes was quite limited due to the following factors: 1) requirement of the absence, or at least depletion, of sense-antisense architectures/overlaps between non-overlapping neighbouring genes; 2) gene partners of non-overlapping neighbouring genes must be located on the opposite DNA strand of the same chromosome; 3) the distance between genes in the non-overlapping neighbouring genes (distance between the 5'-ends or between the 3' ends of representative NM_IDs in a pair) should not be too short (to exclude possible mis-annotated or non-annotated sense-antisense transcripts and false-positive results) or too long (to ensure similar genetic/epigenetic microenvironment for studied controls). In our study, we chose the distance of 0.5 -100 kb between the borders of the non-overlapping neighbouring gene partners. The selection of ‘globally’ identified gene partners from distant loci or chromosomes ("non-neighbouring" gene pairs) would not be sufficient to model the effects of ‘local’ factors in the study of 73-SAGPs effects. Contribution of the alternative local factors was implemented in our workflow to model the appropriate non-overlapping neighbouring genes control sets (Figure S3).

Next, another negative control set of gene pairs was selected: 1) for the set of 2473 PNNGs (Figure S3), we selected 334 non-redundant non-overlapping neighbouring genes (distance between the non-overlapping gene neighbours was the shortest distance within the set, and it varied from 0.5 kb to 87 kb), and performed correlation analysis exactly as it was described for the screening of 73-SAGPs in Figure S1. The procedure resulted in a set of 51 reproducibly correlated pairs of nearest non-overlapping neighboring genes (**PNGs**) (Figure S4). The significantly higher number of 73-SAGPs (73 from 334 SAGPs) obtained by the same procedure and from the same BC datasets than the number of PNGs (51 from 334 PNNGs) (Fisher test,

one-sided p<0.02) indicates the higher connectivity between the gene partners in 73-SAGPs than in PNGs.

Survival prognosis via Data Driven Grouping (DDg) Analysis

The Rotated 2D Data-Driven grouping (2D RDDg)

Briefly, X - and Y- axes rotations were performed in order to find more optimal partition of patients into high risk and low risk subgroups for each pair of Affymetrix probesets.

The rotated 2D Data-Driven grouping (2-D RDDg) is a generalization of the 2-D DDg [48] that considers patients' grouping using different angles for separating the data. In other words, the original X, Y axes are iteratively rotated by angle α (Figure 3B), without losing their orthogonallity property, and in each rotation the patients are grouped similarly as in 2-D DDg (Figure S6) where each design is divided into two sub-designs (Figure 3A and 3C).

The best grouping is the one that minimizes the Wald P value of the β coefficient of the Cox proportional model. The algorithm runs with the corresponding R function.

Note that instead of rotating (transforming) the data by using trigonometric functions:

$$\begin{bmatrix} X' \\ Y' \end{bmatrix} = \begin{bmatrix} X \\ Y \end{bmatrix} \times \begin{bmatrix} \cos(\alpha) & -\sin(\alpha) \\ \sin(\alpha) & \cos(\alpha) \end{bmatrix}$$

where X′, Y′ and X, Y denote the new and the old coordinates, respectively, we rotate the axes themselves (Figure 3B). Analytically, the algorithm works as follows:

1. Assume probesets ID list of size *n* that used to form all possible pairs. For each pair we seek the optimal patient partition into two groups. For probesets $i = 1$ and $j = 2$ ($i = 1, \ldots, N\text{-}1$; $j = i+1$,

…, N), form the candidate cutoffs vectors $\vec{w}^i = y_i^*$ and $\vec{w}^j = y_j^*$ of dimension 1 x Q each, where y_i^* is the log-transformed intensities within (q_{10}^i, q_{90}^i) and Q is the size of $\vec{w}^i$. Each element of the $\vec{w}^i$ is a trial cutoff value (see 1D data-driven function below).

2. Find the cut-off values $\vec{w}^i$ that produce the global minimum P value of the 1-D data-driven grouping and the respective 10 best local minimum P values. Estimate the same for $\vec{w}^i$.

3. For the first element of the "filtered" $\vec{w}^i$ as $\vec{w}_z^i$, $z_i = 1$ and $z_j = 1$, evaluate the prognostic significance of pair i, j for the cutoffs $(\vec{w}_z^i, \vec{w}_z^j)$ by model (1) and by each of the seven designs of Figure 3A.

$$\log h_k^{ij}\left(t_k \middle| x_k^{ij}, \beta_{ij}\right) = \alpha_{ij}(t_k) + \beta_{ij} x_k^{ij} \quad , \qquad (1)$$

where k = 1, …, K are the number of patients and x^{ij} is a dichotomous variable (specified in steps 2-3), indicating the patients groups.

4. Iterate for the combinations of $\vec{w}^i$ and $\vec{w}^j$cutoffs.

5. Rotate each of the X, Y axes by angle α (default is $\alpha = \pi/32$). The rotation works as follows:

i) Denote by $\tan(\vec{\alpha})$ the tan transformation of a vector of angles [0, π]. Note that tan(π/2) = 1.63E+16 (approximated by R function *tan*). Without loss of generality assume that $size(\vec{\alpha}) = S$.

ii) For each combination of cut-offs and each α, estimate $b_0 = c^j + \tan(\alpha_s)\times c^i$ giving new X axis $X' = b_0 - \tan(\alpha_s)\times Y$ and $b_1 = c^j - \tan(\alpha_s)\times c^i$ giving new Y axis $Y' = b_1 - \tan(\alpha_s)\times X$ (s = 1, …, S).

iii) Repeat steps 2-3 and 4i, ii for all tan(α) and cut-off combinations.

6. Provided that the assumptions of model (1) are satisfied, the best cutoff pairs, rotation angle and grouping scheme are selected as follows:

i) At significance level α (typically α = 5%), select the design(s) with number of significant 2-D cut-offs and rotation angles higher than 15%, that is approximately (100 × (# of unique designs))/100.

ii) Among all combinations of (i), select the rotation angle(s) with number of significant 2D cut-offs higher than (100 × (# of unique angles))/100.

iii) All cut-off values of the selected rotation angle(s) and design(s) are significant and kept in a separate file. The respective 2-D P values are adjusted by FDR. The respective statistics of the angle/design combination with most alternative significant cut-offs are kept. Among them, the one with minimum FDR adjusted 2-D P value is stored in a separate file.

7. Iterate 1-3 for all i and j combinations of the N genes ($i = 1, \ldots, N - 1$, $j = i + 1, \ldots, N$).

After calculation of 2D RDDg Wald p-values, we selected pairs of Affyprobesets (representing the host genes-partners of corresponding SAGPs) that are survival significant in both total groups of patients of the Uppsala and Stockholm cohorts and at the same time showing an effect of synergy. General selection workflow is shown in Figure S5. In the 2-D RDDg the rotation angle (coefficient β) have been chosen as the optimally best (the lowest Wald p-value) for both Stockholm and Uppsala cohorts of patients (Figure 3, Table S8). Therefore, Affymetrix probesets which were highly survival significant in one of the two cohorts but not significant in another cohort, have been excluded.

Weighted Voting Grouping (WVG)

The multivariate voting algorithm derives a small pair gene signature that is able to separate the patients into two or more significantly different risk groups. It takes into account the grouping information across several independent pairs. Each 2-D RDDg survival significant pair provides a patients' grouping scheme that may or may not be similar to the others. Ideally, we wish to combine the groupings of several significant features into a composite, final grouping and show that it is still able to separate patients into two distinct disease risk groups. To do this we:

1. Select the g significant features of the list, sorted by the 2-D P value in ascending order. Assign to each pair g the weight $w_g = \frac{-log_{10}p_g}{\sum_{g=1}^{G} -log_{10}p_g}$, where p_g is the 2-D P value of pair g, G is the total number of significant pairs, the transformation of p_g into $-log_{10}p_g$ gives more weight to the low 2D P values (most significant pairs) and $\sum_{g=1}^{G} w_g = 1$.
2. For each g calculate the group indices $x_g^{(k)} \times w_g$ which is a weighted grouping for each patient *k*. Note that $x_g^{(k)}$ takes values 1 (low-risk) or 2 (high-risk).
3. For each patient *k* and G^* = 3, …, G estimate the summary weighted group for each patient $S_k = \sum_{g=1}^{G^*} x_g^{(k)} \times w_g$ and run the 1-D DDg to find the cut-off that maximizes the separation of the low-risk and high risk survival curves. This cut-off determines the patient grouping of the weighted voting.
4. The best signature is the one involving G^* pairs that minimize the P value of 1-D DDg (step 3).

Survival analysis in 73-SAGPs

Application of the 1-D DDg procedure [48] to the 146 genes belonging to 73-SAGPs revealed 32 Affymetrix probesets representing 27 individual host genes associated with survival (Wald p-value <0.05) in the Stockholm and Uppsala cohorts (Table S5) [2]. Current literature analyses showed strong evidence for an association with cancer for 13 genes out of 27 (Table S6). Eight genes have been proposed as potential markers for radiotherapy and chemotherapy sensitivity or for tumour progression *(BCCIP*, *C11orf48*, *CCNE2*, *CYB561D2*, *DBF4*, *EME1*, *TCP1* and *TOX4*). Three of them were reported previously as breast cancer-associated markers: *CCNE2*, a potential biomarker in lymph node-negative breast cancers; *BCCIP*, an important cofactor of *BRCA2* in tumour suppression; *STUB1* is a tumor suppressor in breast cancer that inhibits metastasis by degrading oncogenes, such as *SRC-3* and *ERalpha*.

Microarray analysis

An Agilent 2100 bio analyser was used to verify the quality of isolated total RNA. All the RNA samples used for microarray studies had RIN values above 8, indicating good RNA quality. The GeneChip 3' In vitro transcription (IVT) protocol, which includes reverse transcription to synthesise First strand cDNA, Second-strand cDNA, Biotin-modified mRNA labelling, mRNA purification and fragmentation, were performed according to the manufacturer's protocol. A total of 500 ng of RNA was used for above procedures. Positive control RNA provided by the manufacturer was included for quality control. Hybridisation, washing, and staining of the arrays were carried out as outlined in the GeneChip® Expression Technical Manual. Sixty-two Affymetrix GeneChip® Human Genome U133 Plus 2.0 oligonucleotide chips were used for gene expression analysis. Hybridisation was carried out for 16 h; washing and staining were performed using the Affymetrix Fluidics Station 450 workshop. Probe arrays were scanned using

the Affymetrix GeneChip Scanner 3000, covering 47,000 transcript variants containing over 38,500 function-known genes based on databases (GenBank, dbEST, RefSeq, UniGene database (Build 159 January 25 2003), the Washington University EST trace repository, and the NCBI human genome assembly (Build 31)).

Spliceosome genes as potential drug targets for BC

We analysed the literature regarding the 26 spliceosomal genes identified by the SAGS that were robustly overexpressed in the HR subgroups (Table S9B and Figure 6) .

SF3B5 and *SF3B3* belong to the same SF3b protein complex as an important specific sub-component of the spliceosome, U2-snRNP. The SF3b complex is particularly interesting because it is a promising anticancer drug target[49]. Spliceostatin A (FR901464) is a potent, natural antitumor product that binds to the SF3b complex and inhibits pre-mRNA splicing *in vitro* and *in vivo*. An analogue of FR901464, meayamycin, is a highly effective anti-proliferative agent against human MCF-7 BC cells. Consequently, specific splicing changes induced by SSA can down-regulate genes that are important for cell division, including Cyclin A2 and Aurora A kinase. This mechanism could explain the anti-proliferative effects of SSA[50]. The anti-spliceosome drug, E7107, entered phase I clinical trials for thyroid cancer, and it resulted in stable disease or delayed disease progression in a subset of patients[51]. Other potential drug targets in BC are the members of the ancient family of Sm and like-Sm (LSm) RNA-binding proteins, *LSM3*, *LSM4* and *LSM7*, as was shown for *LSM1*[52]. The spliceosomal heteroheptameric Sm complex is overexpressed in malignant breast tumours compared to benign lesions [53, 54]. Interestingly, the Sm complex defines events that occur during telomerase RNA biogenesis[55]. U2-snRNP-related splicing factors *RBM17/SPF45* [56] and *SF3B1* [57])

represent potential biomarkers of multidrug resistance. Recently, the whole spliceosome was proposed as a target for anticancer treatment in BC [54]. The authors suggested that targeting the deregulated spliceosome core machinery can block mTOR and induce autophagy in cancer cells [54]. Our data indicate that a specific splicing cycle stage, the precatalytic stage, or complex B, might be affected in the high risk subgroup identified by the SAGS. The naturally occurring bioflavonoid isoginkgetin, which inhibits spliceosome complex B splicing *in vitro* [58], is another potentially intriguing drug for BC patients.

Another important property of most anti-spliceosome drugs is their highly selective tumor cytotoxicity[59]. A substantial challenge for the application of novel, promising anti-spliceosome drugs is the identification of the specific subsets of tumours that will be susceptible to splicing-inhibition therapy[60]. One could suggest that the transient, short term pre-treatment of "spliceosome-enriched" breast tumors with drugs specifically targeting the spliceosome may not lead to substantial drug side effects, though it could potentially lead to a significant increase of tumor sensitivity to the subsequent course of standard chemo/hormonal therapy. In this context, anti-spliceosome drugs could be a promising alternative to inhibit cell cycle progression and tumor growth in breast tumors *enriched with deregulated (overexpressed) spliceosome genes*. Specific trial studies in patient subgroups identified by the SAGS could provide the clues to resolve this challenge.

Definitions

<u>Coding-Coding Cis-Sense-Antisense Gene Pair</u> (**SAGP**) is a complex transcriptional architecture containing a pair of protein-encoding host genes on opposite strands of a chromosome sharing

common (overlapping) region. It also includes all other transcripts (with SA overlaps or without) on both DNA strands within the common span of these two genes.

BC-relevant SAGPs (**73-SAGPs**) are the SAGPs which show reproducible significant correlations between mRNAs of their sense and antisense host genes-partners in BC cells as well as significant changes in their overall correlation profile in breast tumors as compared with normal breast tissue.

The term Statistical Partition Model (**SPM**) defines the cut-off values of gene expression level values (low or high) and other necessary parameters (e.g., partition design, rotation angle) for a gene or a gene pair in a given group of tumor samples (obtained from distinct patients) and stratifies them into subgroups with a high-risk- and a low-risk of a potentially fatal medical condition.

The method of Composite Survival Prognosis (**CSP**) refers to the group of prognostic methods which integrate the information for individual features (e.g., genes or gene pairs) into a significantly improved integrated partition. CSP includes, but not limited to, Weighted Voting Grouping (WVG), Hierarchical Clustering Analysis (HCA) and Principal Component Analysis (PCA).

The 1-Dimensional Data-Driven grouping (**1-D DDg method**) associates survival events (DFS) to single gene expression data available for a set of patients *K* suffering from a medical condition using survival analysis with the Cox proportional hazards model.

The 2-Dimensional Data-Driven grouping (**2-D DDg method**) is an extension of the 1-D DDg method. It assumes that in some situations the expression values of individual genes could be

organized in 2-dimensional space as gene pairs, which may provide more statistically significant partition model of the survival prognosis, than the individual genes used for patient' grouping independently.

2-Dimensional Rotated Data-Driven grouping (**2-D RDDg method**) is the generalized 2-D DDg method. It assumes that in certain situations using 2-D DDg method may not be the most optimal solution for statistical partition of patients with a medical condition (e.g., when pairs of genes are significantly correlated). In 2-D RDDg expression level values for two genes of the same gene pair, expressed as points in a two-dimensional space are compared to perpendicular cut-off lines which are iteratively rotated in the two directions without losing their orthogonallity property to improve the quality of a statistical partition.

Weighted Voting Grouping (**WVG method**) can be defined as a statistical method of composite survival prognosis (CSP) integrating the information for individual features (genes or gene pairs) into a significantly improved integrated partition. CSP methods may also include, but are not limited to, Hierarchical Clustering Analysis (HCA) and Principal Component Analysis (PCA).WVG can be used for the integration of the data obtained by either 1-D DDg, 2-D DDg or 2-D RDDg methods for individual features for patients partition (genes, gene pairs) into a dramatically improved integrated partition. The outputs from a plurality of individual features are combined as a weighted sum, using weights indicative of the relative quality of the statistical partition models (e.g. as measured by the Wald p-values) in relation to the medical condition (such as BC). The weighted sum is then compared to a threshold, which itself may be optimized by seeking a value with maximal statistical significance.

Synergistic SAGPs are the SAGPs in which each of gene partners has lower prognostic power in survival analysis (e.g., higher 1-D DDg p-value) as compared with the case when they are analysed together (e.g., lower 2-D RDDg p-value) in survival analysis.

Synergistic Survival Significant SAGP is the SAGP which exhibit interaction effect in comparison with each of the gene forming the gene pair Wald p-value for <0.05.

Nearest Genes-Neighbours (**NGNs**) are the nearest upstream or nearest downstream neighbouring genes for the studied 73-SAGP (see detailed description in the TextS1). Used as a negative control in correlation analyses of SAGPs and in proximal promoter analysis.

A pair of Non-Overlapping Neighbouring Genes is the paired genes which are distinct from NGNs in a sense that they are neighbouring to each other, but not to the gene-partners of a SAGP from 73-SAGPs set as well as do not have RefSeq/RefSeq SA overlaps. Minimal distance between the nearest borders of their genes has been arbitrary set as varying from 0.5 to 100 kb; they comprise the gene pairs sets used as negative controls in survival, correlation and proximal promoters analyses.

A Set of Pairs of Reproducibly Correlated Non-Overlapping Neighbouring Genes (**PNGs**) - the subset of Non-Overlapping Neighbouring Genes (n=51) selected by the criterion of significant correlation in 3 independent breast cancer cohorts and 2 BC subgroups (p<0.05), in the same way as 73-SAGPs (Figures S1 and S4). Used as a negative control in proximal promoter analysis.

Sense-Antisense Gene Signature (**SAGS**) is comprised of twelve synergistic survival significant SAGPs.

Kolmogorov-Smirnov test **(KS test)**.

Disease Free Survival (**DFS**).

Supplementary References:

1. Graham K, de las Morenas A, Tripathi A, King C, Kavanah M, Mendez J, Stone M, Slama J, Miller M, Antoine G, Willers H, Sebastiani P and Rosenberg CL. Gene expression in histologically normal epithelium from breast cancer patients and from cancer-free prophylactic mastectomy patients shares a similar profile. British journal of cancer. 2010; 102(8):1284-1293.
2. Ivshina AV, George J, Senko O, Mow B, Putti TC, Smeds J, Lindahl T, Pawitan Y, Hall P, Nordgren H, Wong JE, Liu ET, Bergh J, Kuznetsov VA and Miller LD. Genetic reclassification of histologic grade delineates new clinical subtypes of breast cancer. Cancer research. 2006; 66(21):10292-10301.
3. Pawitan Y, Bjohle J, Amler L, Borg AL, Egyhazi S, Hall P, Han X, Holmberg L, Huang F, Klaar S, Liu ET, Miller L, Nordgren H, Ploner A, Sandelin K, Shaw PM, et al. Gene expression profiling spares early breast cancer patients from adjuvant therapy: derived and validated in two population-based cohorts. Breast Cancer Res. 2005; 7(6):R953-964.
4. Rewari A, Lu H, Parikh R, Yang Q, Shen Z and Haffty BG. BCCIP as a prognostic marker for radiotherapy of laryngeal cancer. Radiother Oncol. 2009; 90(2):183-188.
5. Liu J, Yuan Y, Huan J and Shen Z. Inhibition of breast and brain cancer cell growth by BCCIPalpha, an evolutionarily conserved nuclear protein that interacts with BRCA2. Oncogene. 2001; 20(3):336-345.
6. Chen L, Chen W, Zhao L, Yu HZ and Li X. Immunoscreening of urinary bladder cancer cDNA library and identification of potential tumor antigen. World journal of urology. 2009; 27(1):107-112.
7. Cadinanos J, Llorente JL, de la Rosa J, Villameytide JA, Illan R, Duran NS, Murias E and Cabanillas R. Novel germline SDHD deletion associated with an unusual sympathetic head and neck paraganglioma. Head & neck.
8. Han S, Lee KM, Park SK, Lee JE, Ahn HS, Shin HY, Kang HJ, Koo HH, Seo JJ, Choi JE, Ahn YO and Kang D. Genome-wide association study of childhood acute lymphoblastic leukemia in Korea. Leukemia research. 2010; 34(10):1271-1274.
9. Sieuwerts AM, Look MP, Meijer-van Gelder ME, Timmermans M, Trapman AM, Garcia RR, Arnold M, Goedheer AJ, de Weerd V, Portengen H, Klijn JG and Foekens JA. Which cyclin E prevails as prognostic marker for breast cancer? Results from a retrospective study involving 635 lymph node-negative breast cancer patients. Clin Cancer Res. 2006; 12(11 Pt 1):3319-3328.
10. Faghihi MA, Kocerha J, Modarresi F, Engstrom PG, Chalk AM, Brothers SP, Koesema E, St Laurent G and Wahlestedt C. RNAi screen indicates widespread biological function for human natural antisense transcripts. PloS one. 5(10).
11. Ohtani S, Iwamaru A, Deng W, Ueda K, Wu G, Jayachandran G, Kondo S, Atkinson EN, Minna JD, Roth JA and Ji L. Tumor suppressor 101F6 and ascorbate synergistically and selectively inhibit non-small cell lung cancer growth by caspase-independent apoptosis and autophagy. Cancer research. 2007; 67(13):6293-6303.
12. Bivi N, Romanello M, Harrison R, Clarke I, Hoyle DC, Moro L, Ortolani F, Bonetti A, Quadrifoglio F, Tell G and Delneri D. Identification of secondary targets of N-containing bisphosphonates in mammalian cells via parallel competition analysis of the barcoded yeast deletion collection. Genome biology. 2009; 10(9):R93.

13. Bonte D, Lindvall C, Liu H, Dykema K, Furge K and Weinreich M. Cdc7-Dbf4 kinase overexpression in multiple cancers and tumor cell lines is correlated with p53 inactivation. Neoplasia (New York, NY. 2008; 10(9):920-931.
14. Tomoda Y, Katsura M, Okajima M, Hosoya N, Kohno N and Miyagawa K. Functional evidence for Eme1 as a marker of cisplatin resistance. International journal of cancer. 2009; 124(12):2997-3001.
15. Kajiro M, Hirota R, Nakajima Y, Kawanowa K, So-ma K, Ito I, Yamaguchi Y, Ohie SH, Kobayashi Y, Seino Y, Kawano M, Kawabe Y, Takei H, Hayashi S, Kurosumi M, Murayama A, et al. The ubiquitin ligase CHIP acts as an upstream regulator of oncogenic pathways. Nature cell biology. 2009; 11(3):312-319.
16. Fan M, Park A and Nephew KP. CHIP (carboxyl terminus of Hsc70-interacting protein) promotes basal and geldanamycin-induced degradation of estrogen receptor-alpha. Molecular endocrinology (Baltimore, Md. 2005; 19(12):2901-2914.
17. Macleod K, Mullen P, Sewell J, Rabiasz G, Lawrie S, Miller E, Smyth JF and Langdon SP. Altered ErbB receptor signaling and gene expression in cisplatin-resistant ovarian cancer. Cancer research. 2005; 65(15):6789-6800.
18. Moparthi SB, Arbman G, Wallin A, Kayed H, Kleeff J, Zentgraf H and Sun XF. Expression of MAC30 protein is related to survival and biological variables in primary and metastatic colorectal cancers. International journal of oncology. 2007; 30(1):91-95.
19. Puch CB, Barbier E, Kraut A, Coute Y, Fuchs J, Buhot A, Livache T, Seve M, Favier A, Douki T, Gasparutto D, Sauvaigo S and Breton J. TOX4 and its binding partners recognize DNA adducts generated by platinum anticancer drugs. Archives of biochemistry and biophysics. 2011.
20. Gong Y, Duvvuri M, Duncan MB, Liu J and Krise JP. Niemann-Pick C1 protein facilitates the efflux of the anticancer drug daunorubicin from cells according to a novel vesicle-mediated pathway. The Journal of pharmacology and experimental therapeutics. 2006; 316(1):242-247.
21. Hutterer A, Berdnik D, Wirtz-Peitz F, Zigman M, Schleiffer A and Knoblich JA. Mitotic activation of the kinase Aurora-A requires its binding partner Bora. Developmental cell. 2006; 11(2):147-157.
22. Niu N, Qin Y, Fridley BL, Hou J, Kalari KR, Zhu M, Wu TY, Jenkins GD, Batzler A and Wang L. Radiation pharmacogenomics: a genome-wide association approach to identify radiation response biomarkers using human lymphoblastoid cell lines. Genome research. 2010; 20(11):1482-1492.
23. Rozenblum E, Vahteristo P, Sandberg T, Bergthorsson JT, Syrjakoski K, Weaver D, Haraldsson K, Johannsdottir HK, Vehmanen P, Nigam S, Golberger N, Robbins C, Pak E, Dutra A, Gillander E, Stephan DA, et al. A genomic map of a 6-Mb region at 13q21-q22 implicated in cancer development: identification and characterization of candidate genes. Human genetics. 2002; 110(2):111-121.
24. Liang L, Qu L and Ding Y. Protein and mRNA characterization in human colorectal carcinoma cell lines with different metastatic potentials. Cancer investigation. 2007; 25(6):427-434.
25. Lim J, Kuroki T, Ozaki K, Kohsaki H, Yamori T, Tsuruo T, Nakamori S, Imaoka S, Endo M and Nakamura Y. Isolation of murine and human homologues of the fission-yeast dis3+ gene encoding a mitotic-control protein and its overexpression in cancer cells with progressive phenotype. Cancer research. 1997; 57(5):921-925.
26. Chang SH, Chung YS, Hwang SK, Kwon JT, Minai-Tehrani A, Kim S, Park SB, Kim YS and Cho MH. Lentiviral vector-mediated shRNA against AIMP2-DX2 suppresses lung cancer cell growth through blocking glucose uptake. Molecules and cells. 2012; 33(6):553-562.
27. Choi JW, Kim DG, Lee AE, Kim HR, Lee JY, Kwon NH, Shin YK, Hwang SK, Chang SH, Cho MH, Choi YL, Kim J, Oh SH, Kim B, Kim SY, Jeon HS, et al. Cancer-associated splicing variant of tumor suppressor AIMP2/p38: pathological implication in tumorigenesis. PLoS genetics. 2011; 7(3):e1001351.
28. Wang Y, Wang Y, Liu Q, Xu G, Mao F, Qin T, Teng H, Cai W, Yu P, Cai T, Zhao M, Sun ZS and Xie C. Comparative RNA-seq analysis reveals potential mechanisms mediating the conversion to androgen independence in an LNCaP progression cell model. Cancer letters. 2014; 342(1):130-138.

29. Komlosi V, Hitre E, Pap E, Adleff V, Reti A, Szekely E, Biro A, Rudnai P, Schoket B, Muller J, Toth B, Otto S, Kasler M, Kralovanszky J and Budai B. SHMT1 1420 and MTHFR 677 variants are associated with rectal but not colon cancer. BMC cancer. 2010; 10:525.
30. Al-Sarraf N, Reiff JN, Hinrichsen J, Mahmood S, Teh BT, McGovern E, De Meyts P, O'Byrne K J and Gray SG. DOK4/IRS-5 expression is altered in clear cell renal cell carcinoma. International journal of cancer. 2007; 121(5):992-998.
31. Park JH, Kim NS, Park JY, Chae YS, Kim JG, Sohn SK, Moon JH, Kang BW, Ryoo HM, Bae SH, Choi GS and Jun SH. MGMT -535G>T polymorphism is associated with prognosis for patients with metastatic colorectal cancer treated with oxaliplatin-based chemotherapy. Journal of cancer research and clinical oncology. 2010; 136(8):1135-1142.
32. Joerger M, deJong D, Burylo A, Burgers JA, Baas P, Huitema AD, Beijnen JH and Schellens JH. Tubulin, BRCA1, ERCC1, Abraxas, RAP80 mRNA expression, p53/p21 immunohistochemistry and clinical outcome in patients with advanced non small-cell lung cancer receiving first-line platinum-gemcitabine chemotherapy. Lung cancer (Amsterdam, Netherlands). 2011; 74(2):310-317.
33. Han M, Wang H, Zhang HT and Han Z. The PDZ protein TIP-1 facilitates cell migration and pulmonary metastasis of human invasive breast cancer cells in athymic mice. Biochemical and biophysical research communications. 2012; 422(1):139-145.
34. Terp MG, Lund RR, Jensen ON, Leth-Larsen R and Ditzel HJ. Identification of markers associated with highly aggressive metastatic phenotypes using quantitative comparative proteomics. Cancer genomics & proteomics. 2012; 9(5):265-273.
35. Kim K, Heo K, Choi J, Jackson S, Kim H, Xiong Y and An W. Vpr-binding protein antagonizes p53-mediated transcription via direct interaction with H3 tail. Molecular and cellular biology. 2012; 32(4):783-796.
36. Brauweiler A, Lorick KL, Lee JP, Tsai YC, Chan D, Weissman AM, Drabkin HA and Gemmill RM. RING-dependent tumor suppression and G2/M arrest induced by the TRC8 hereditary kidney cancer gene. Oncogene. 2007; 26(16):2263-2271.
37. Edgren H, Murumagi A, Kangaspeska S, Nicorici D, Hongisto V, Kleivi K, Rye IH, Nyberg S, Wolf M, Borresen-Dale AL and Kallioniemi O. Identification of fusion genes in breast cancer by paired-end RNA-sequencing. Genome biology. 2011; 12(1):R6.
38. Richardson AL, Wang ZC, De Nicolo A, Lu X, Brown M, Miron A, Liao X, Iglehart JD, Livingston DM and Ganesan S. X chromosomal abnormalities in basal-like human breast cancer. Cancer cell. 2006; 9(2):121-132.
39. Li Y, Zou L, Li Q, Haibe-Kains B, Tian R, Li Y, Desmedt C, Sotiriou C, Szallasi Z, Iglehart JD, Richardson AL and Wang ZC. Amplification of LAPTM4B and YWHAZ contributes to chemotherapy resistance and recurrence of breast cancer. Nature medicine. 2010; 16(2):214-218.
40. Sabatier R, Finetti P, Cervera N, Lambaudie E, Esterni B, Mamessier E, Tallet A, Chabannon C, Extra JM, Jacquemier J, Viens P, Birnbaum D and Bertucci F. A gene expression signature identifies two prognostic subgroups of basal breast cancer. Breast cancer research and treatment. 2011; 126(2):407-420.
41. Loi S, Haibe-Kains B, Desmedt C, Lallemand F, Tutt AM, Gillet C, Ellis P, Harris A, Bergh J, Foekens JA, Klijn JG, Larsimont D, Buyse M, Bontempi G, Delorenzi M, Piccart MJ, et al. Definition of clinically distinct molecular subtypes in estrogen receptor-positive breast carcinomas through genomic grade. J Clin Oncol. 2007; 25(10):1239-1246.
42. Loi S, Haibe-Kains B, Desmedt C, Wirapati P, Lallemand F, Tutt AM, Gillet C, Ellis P, Ryder K, Reid JF, Daidone MG, Pierotti MA, Berns EM, Jansen MP, Foekens JA, Delorenzi M, et al. Predicting prognosis using molecular profiling in estrogen receptor-positive breast cancer treated with tamoxifen. BMC genomics. 2008; 9:239.

43. Tripathi A, King C, de la Morenas A, Perry VK, Burke B, Antoine GA, Hirsch EF, Kavanah M, Mendez J, Stone M, Gerry NP, Lenburg ME and Rosenberg CL. Gene expression abnormalities in histologically normal breast epithelium of breast cancer patients. International journal of cancer. 2008; 122(7):1557-1566.
44. Chen DT, Nasir A, Culhane A, Venkataramu C, Fulp W, Rubio R, Wang T, Agrawal D, McCarthy SM, Gruidl M, Bloom G, Anderson T, White J, Quackenbush J and Yeatman T. Proliferative genes dominate malignancy-risk gene signature in histologically-normal breast tissue. Breast cancer research and treatment. 2011; 119(2):335-346.
45. Hu X, Stern HM, Ge L, O'Brien C, Haydu L, Honchell CD, Haverty PM, Peters BA, Wu TD, Amler LC, Chant J, Stokoe D, Lackner MR and Cavet G. Genetic alterations and oncogenic pathways associated with breast cancer subtypes. Mol Cancer Res. 2009; 7(4):511-522.
46. Kapranov P, Willingham AT and Gingeras TR. Genome-wide transcription and the implications for genomic organization. Nature reviews. 2007; 8(6):413-423.
47. Grinchuk OV, Motakis E. and Kuznetsov V.A. Complex sense-antisense architecture of TNFAIP1/POLDIP2 on 17q11.2 represents a novel transcriptional structural-functional gene module involved in breast cancer progression. BMC genomics. 2010; 11(Suppl 1): S9.
48. Motakis E, Ivshina AV and Kuznetsov VA. Data-driven approach to predict survival of cancer patients: estimation of microarray genes' prediction significance by Cox proportional hazard regression model. IEEE Eng Med Biol Mag. 2009; 28(4):58-66.
49. Albert BJ, Sivaramakrishnan A, Naka T, Czaicki NL and Koide K. Total syntheses, fragmentation studies, and antitumor/antiproliferative activities of FR901464 and its low picomolar analogue. Journal of the American Chemical Society. 2007; 129(9):2648-2659.
50. Albert BJ, McPherson PA, O'Brien K, Czaicki NL, Destefino V, Osman S, Li M, Day BW, Grabowski PJ, Moore MJ, Vogt A and Koide K. Meayamycin inhibits pre-messenger RNA splicing and exhibits picomolar activity against multidrug-resistant cells. Molecular cancer therapeutics. 2009; 8(8):2308-2318.
51. Tsimberidou AM, Vaklavas C, Wen S, Hong D, Wheler J, Ng C, Naing A, Tse S, Busaidy N, Markman M, Sherman SI and Kurzrock R. Phase I clinical trials in 56 patients with thyroid cancer: the M. D. Anderson Cancer Center experience. The Journal of clinical endocrinology and metabolism. 2009; 94(11):4423-4432.
52. Kelley JR, Fraser MM, Hubbard JM, Watson DK and Cole DJ. CaSm antisense gene therapy: a novel approach for the treatment of pancreatic cancer. Anticancer research. 2003; 23(3A):2007-2013.
53. Andre F, Michiels S, Dessen P, Scott V, Suciu V, Uzan C, Lazar V, Lacroix L, Vassal G, Spielmann M, Vielh P and Delaloge S. Exonic expression profiling of breast cancer and benign lesions: a retrospective analysis. The lancet oncology. 2009; 10(4):381-390.
54. Quidville V, Alsafadi S, Goubar A, Commo F, Scott V, Durieu C, Girault I, Baconnais S, Lecam E, Lazar V, Delaloge S, Saghatchian M, Pautier P, Morice P, Dessen P, Vagner S, et al. Targeting the deregulated spliceosome core machinery in cancer cells triggers mTOR blockade and autophagy. Cancer research. 2013; 73(7):2247-2258.
55. Tang W, Kannan R, Blanchette M and Baumann P. Telomerase RNA biogenesis involves sequential binding by Sm and Lsm complexes. Nature. 2012; 484(7393):260-264.
56. Sampath J, Long PR, Shepard RL, Xia X, Devanarayan V, Sandusky GE, Perry WL, 3rd, Dantzig AH, Williamson M, Rolfe M and Moore RE. Human SPF45, a splicing factor, has limited expression in normal tissues, is overexpressed in many tumors, and can confer a multidrug-resistant phenotype to cells. The American journal of pathology. 2003; 163(5):1781-1790.
57. Rossi D, Bruscaggin A, Spina V, Rasi S, Khiabanian H, Messina M, Fangazio M, Vaisitti T, Monti S, Chiaretti S, Guarini A, Del Giudice I, Cerri M, Cresta S, Deambrogi C, Gargiulo E, et al. Mutations of the

SF3B1 splicing factor in chronic lymphocytic leukemia: association with progression and fludarabine-refractoriness. Blood. 2011; 118(26):6904-6908.
58. O'Brien K, Matlin AJ, Lowell AM and Moore MJ. The biflavonoid isoginkgetin is a general inhibitor of Pre-mRNA splicing. J Biol Chem. 2008; 283(48):33147-33154.
59. Webb TR, Joyner AS and Potter PM. The development and application of small molecule modulators of SF3b as therapeutic agents for cancer. Drug discovery today. 2012; 18(1-2):43-49.
60. Kaida D, Schneider-Poetsch T and Yoshida M. Splicing in oncogenesis and tumor suppression. Cancer science. 2012; 103(9):1611-1616.
