# Supplementary material for: Sense-antisense gene-pairs in breast cancer and associated pathological pathways
Source: Oncotarget. 2015 Oct 28;6(39):42197–221. doi: 10.18632/oncotarget.6255 (PMC4747219; doi:10.18632/oncotarget.6255)
Supplement: Supplementary file 2 [file oncotarget-06-42197-s002.pdf]

Sense-antisense gene-pairs in breast cancer and associated pathological pathways

Supplementary Material

Supplementary Figure S1A: Schematic strategy for identification of correlated SAGPs in BC patients.

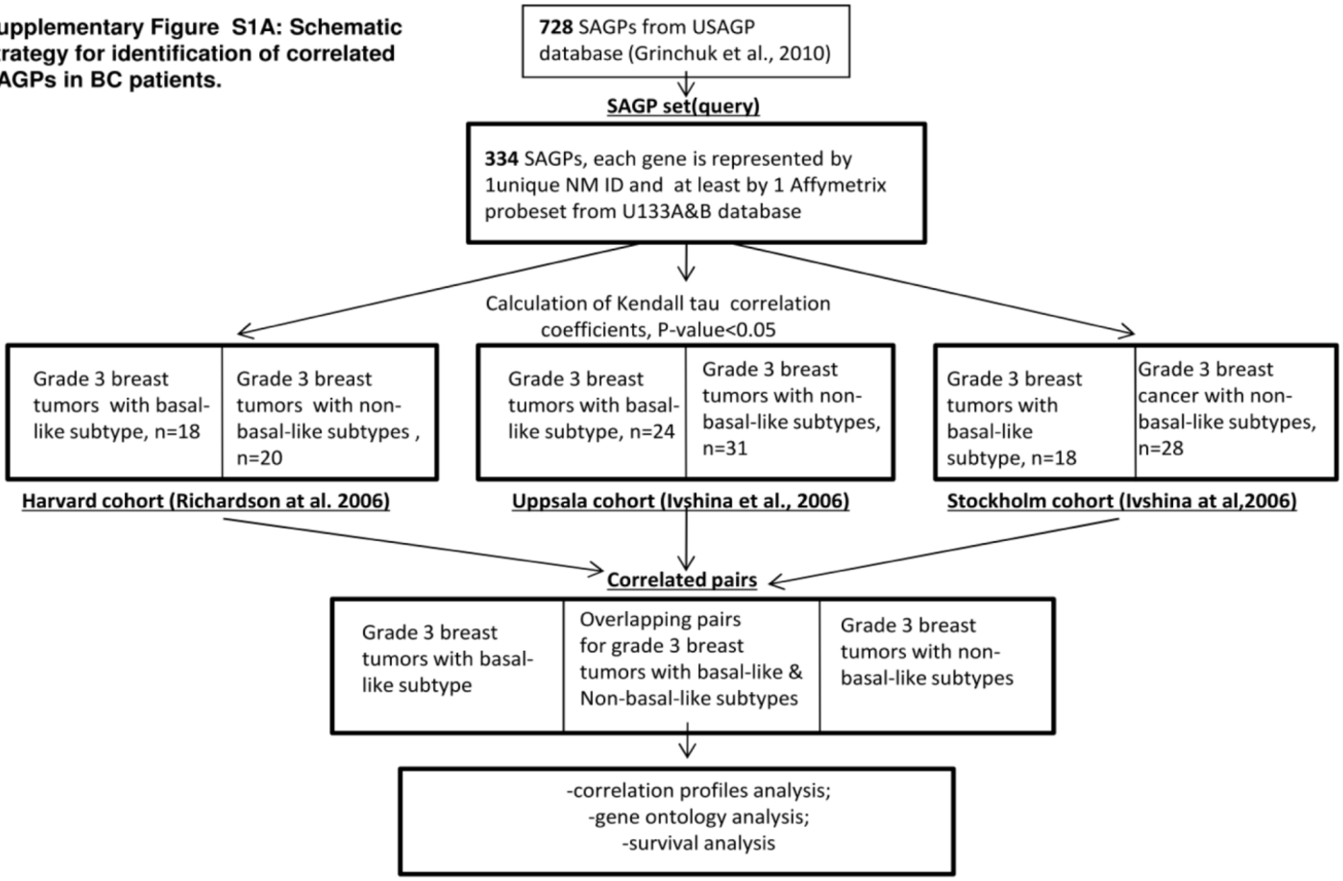

Supplementary Figure S1B: Selection and enrichment analysis of significantly correlated Affymetrix probesets pairs corresponding to common SAGPs found in three breast cancer cohorts. Venn diagrams show the numbers of significantly correlated gene pairs (Kendall tau correlation, p<0.05) at each step of the feature selection procedure.

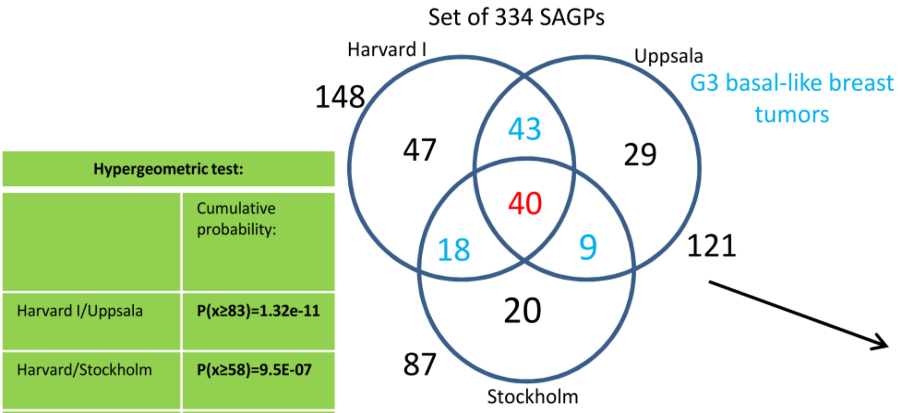

Hypergeometric test:	
	Cumulative probability:
Harvard I/Uppsala	**P(x≥83)=1.32e-11**
Harvard/Stockholm	**P(x≥58)=9.5E-07**
Stockholm/Uppsala	**P(x≥49)=6.8E-06**

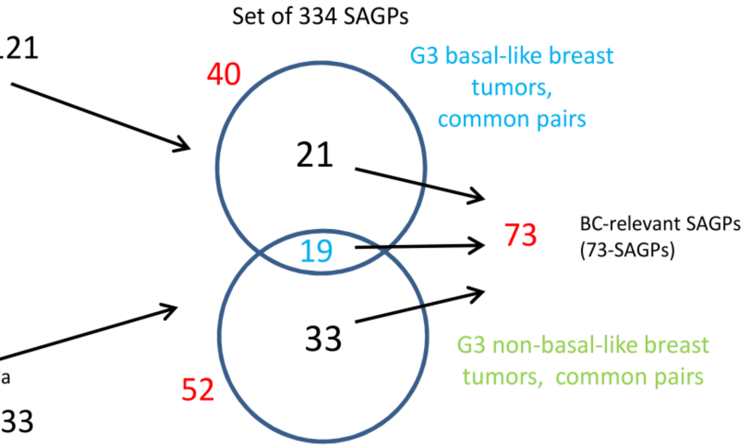

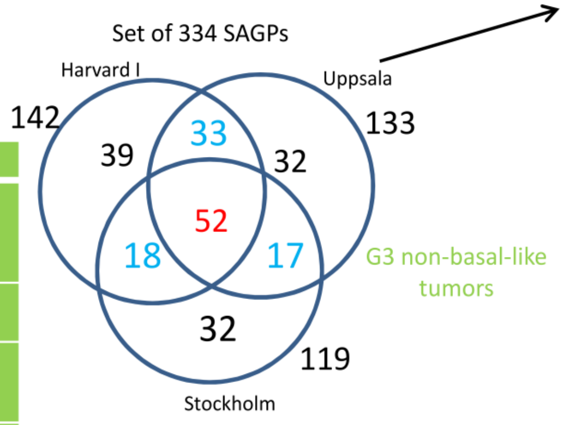

Hypergeometric test:	
	Cumulative probability:
Harvard I/Uppsala	**P(x≥85)=1.1E-10**
Harvard I/Stockholm	**P(x≥70)=6.2E-06**
Stockholm/Uppsala	**P(x≥69)=4.4E-07**

Hypergeometric test:	
	Cumulative probability:
G3 basal-like breast tumors/ G3 non-basal-like breast tumors	**P(x≥19)=2.4E-07**

Supplementary Figure S1C: Differentially expressed genes between the Grade 3 “basal-like” and Grade 3 “non-basal-like” breast tumors subgroups identified in the combined Uppsala and Stockholm BC cohorts. 101 BC patients with Grade 3 tumors were analysed in total. BL: “basal-like”, nBL: “non-basal-like”. Among the 11 significantly up-regulated genes in the “basal-like” tumors 6 are organized in SAGPs: PDSS1/ABI1, BORA/DIS3 and WDR77/ATP5F1. *: the genes down-regulated in “basal-like” breast tumors. X axis: normalized gene expression values (Affymetrix U133A&B platform). All the presented genes passed the t-test with multiple testing correction (Q-value <0.05).

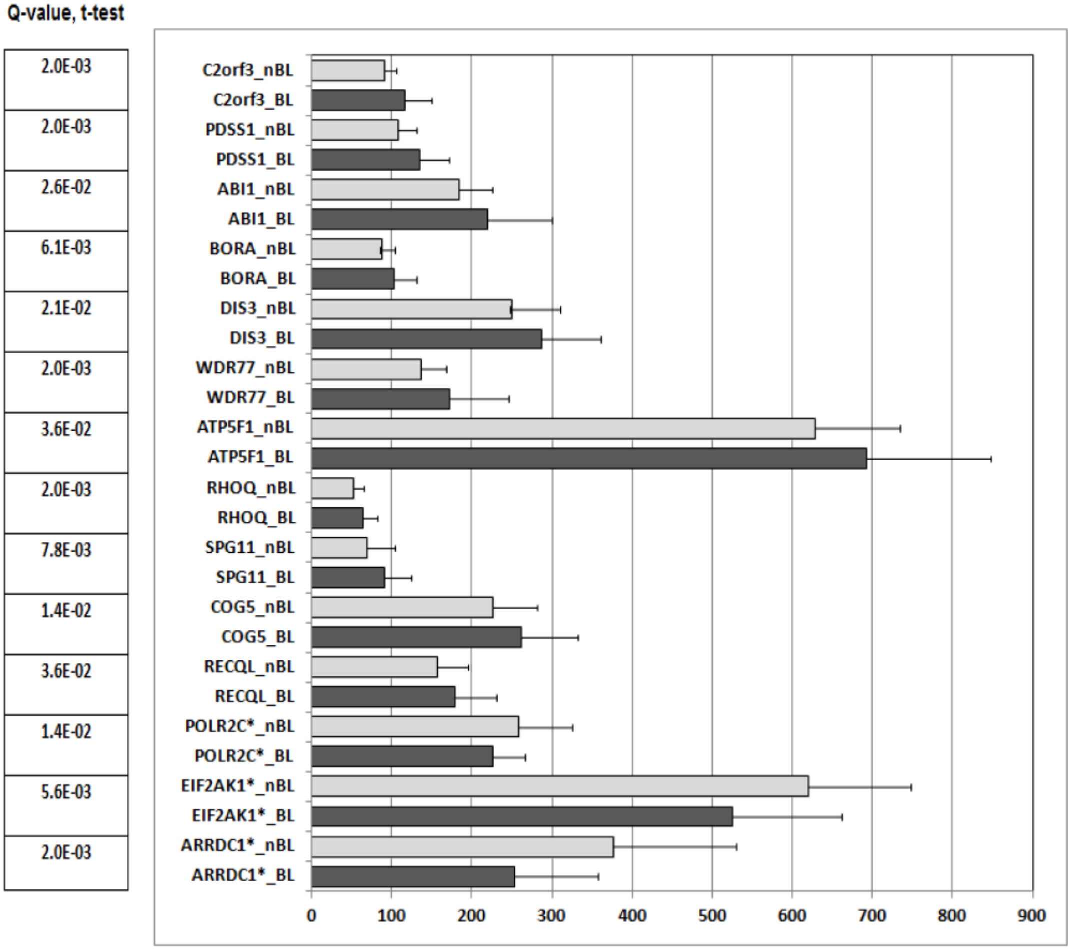

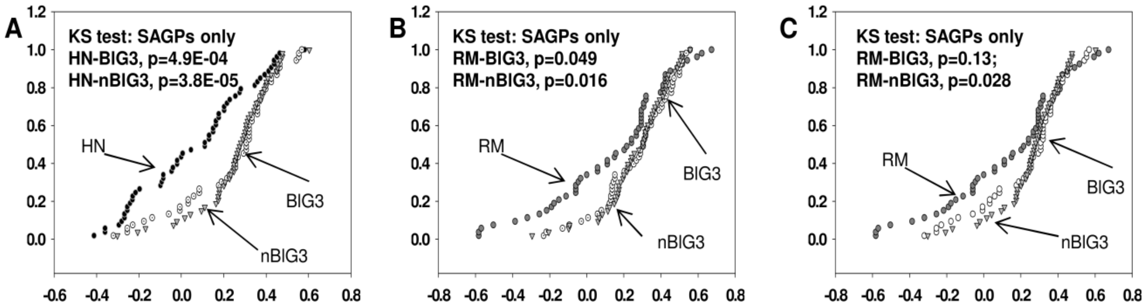

Supplementary Figure S1D: Comparison of cumulative curves of Kendall's Tau correlation coefficients in various gene sets.
For each SAGP, one corresponding, representative pair of Affymetrix probe sets with the strongest Kendall Tau correlation coefficient (positive or negative) was selected. X axis: Kendall Tau correlation coefficient, Y axis: cumulative relative frequency. (**A)** Cumulative curves for 53 SAGPs in G3 "basal-like" (BlG3, white circles), G3 "non-basal-like" (nBlG3, light grey triangles) from the Stockholm BC cohort and histologically normal breast epithelium samples (HN, dark grey circles). (**B** and **C)** Cumulative curves for 53 SAGPs in BlG3 (white circles), nBlG3 (light grey triangles) in the Uppsala (**B**) and Stockholm (**C**) BC cohorts and prophylactic reduction mastectomy samples (RM, dark grey circles).

Supplementary Figure S2: DNA CNVs for gene partners of 73-SAGPs and their co-localized NGNs. White and black bars correspond to the average CNV value per gene for upstream and downstream gene partners of ccSAGPs, light blue and light green bars - to the average CNV values per gene of their nearest upstream and downstream genes-neighbors, respectively. Black lines mark the accepted interval (2±0.25) for normal DNA CNV values. (**A** and **C**) DNA CNVs in 38 breast cancer cell lines ; (**B** and **D)** DNA CNVs in 93 primary breast tumors. DNA CNV for the oncogenes *ERBB2* and *MYC* known to be amplified in BC are shown for comparison.

A.

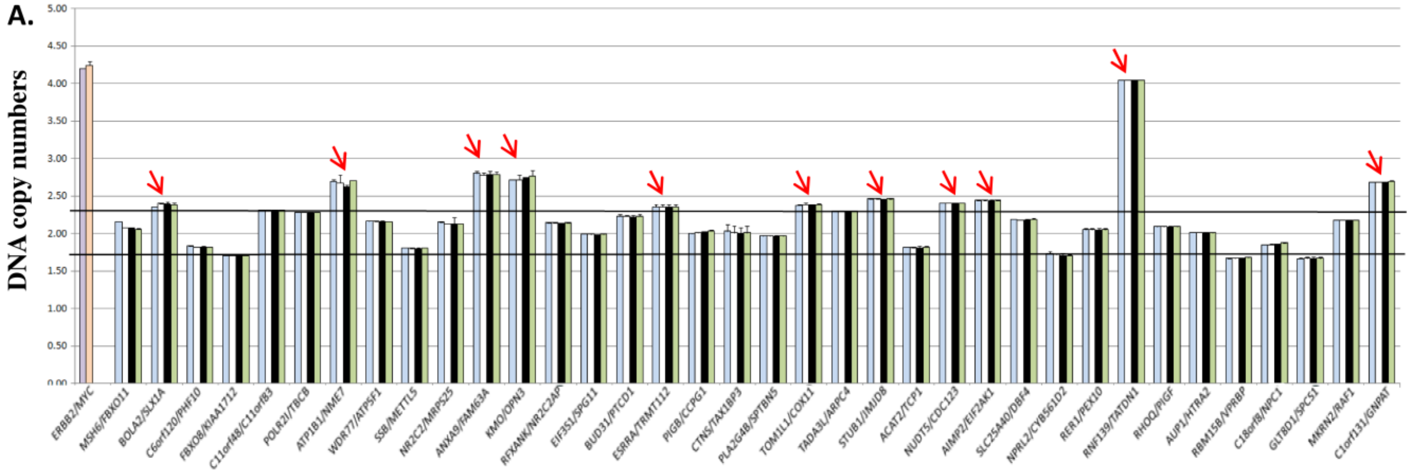

B.

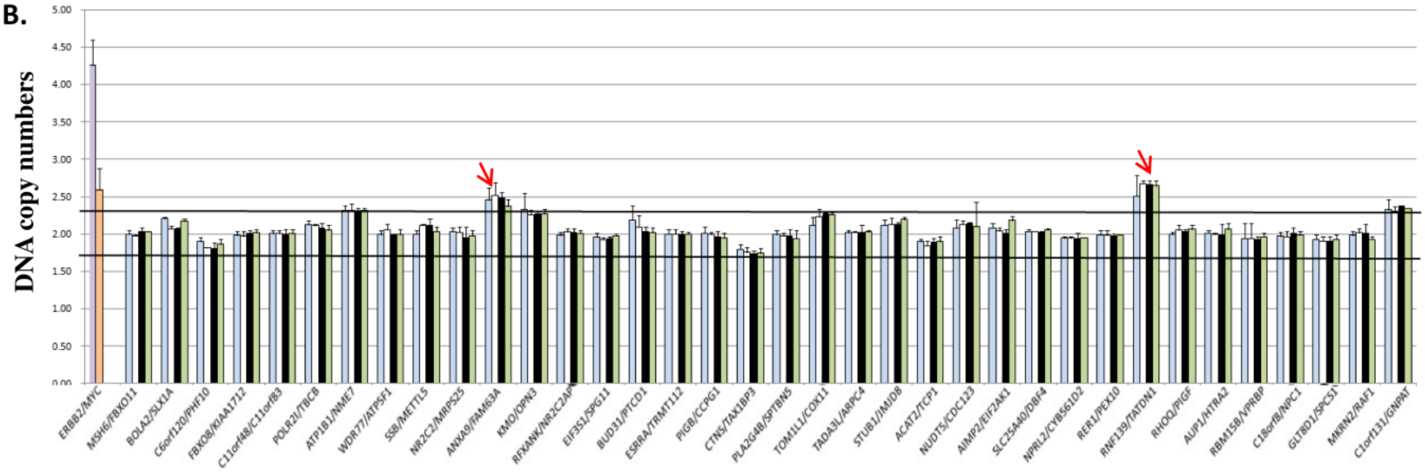

Supplementary Figure S2 (Continued)

C.

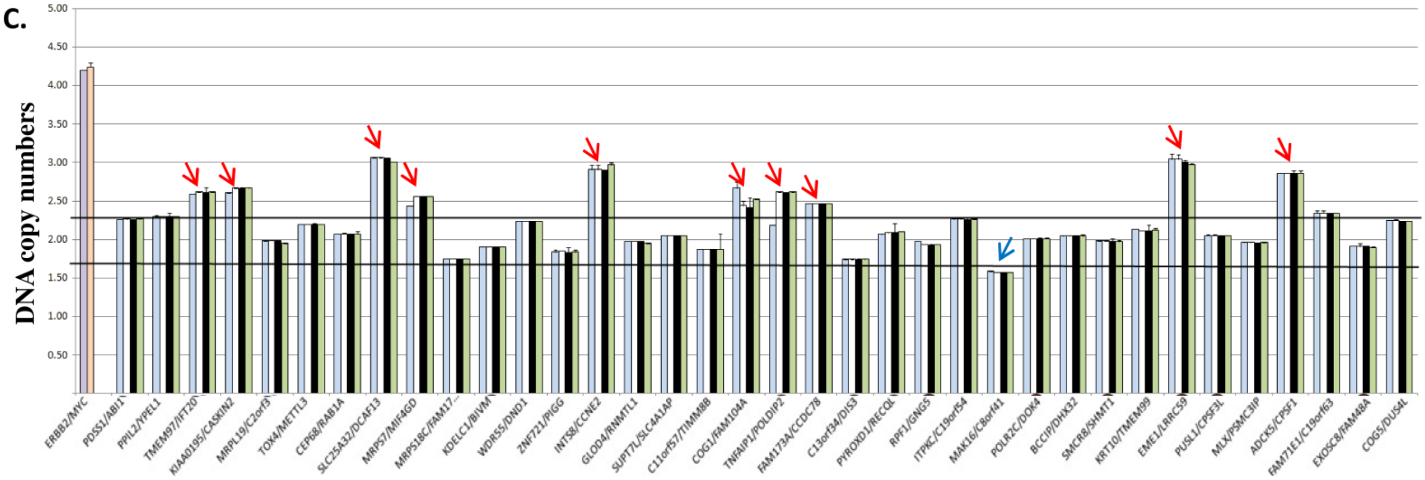

D.

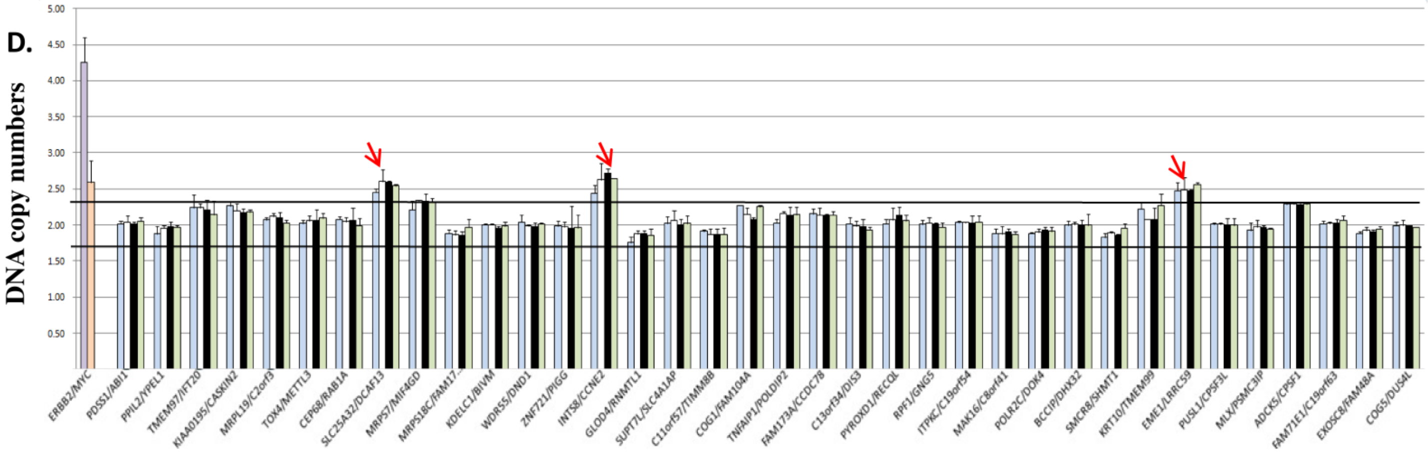

Supplementary Figure S3: The workflow for the isolation of the reproducibly correlated PNGs.

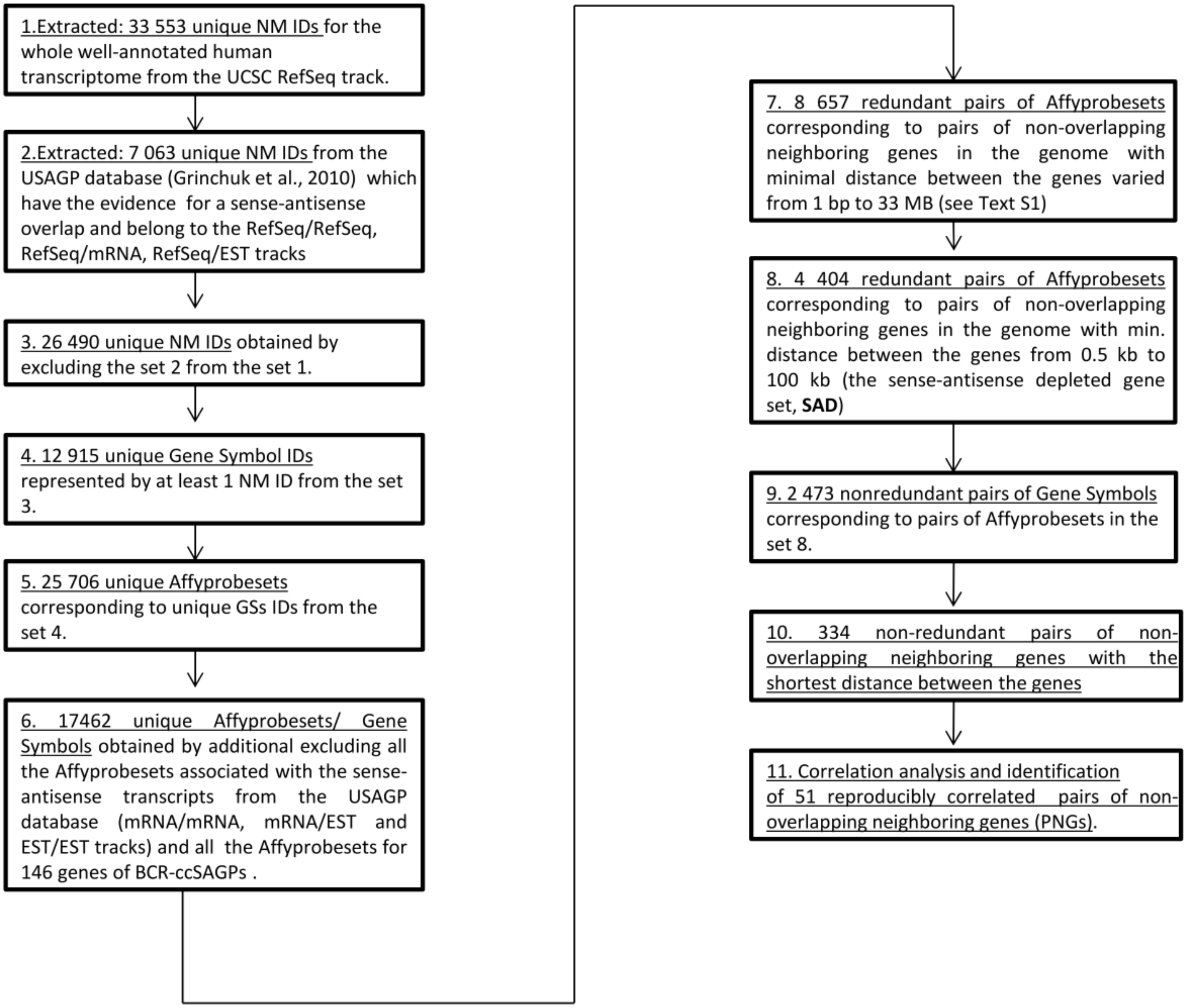

Supplementary Figure S4: Selection and enrichment analysis of significantly correlated Affymetrix probe sets pairs corresponding to the PNGs in three BC cohorts. Venn diagrams show the numbers of significantly correlated pairs of non-overlapping neighboring genes (Kendall tau correlation, p<0.05) at each step of gene expression data processing.

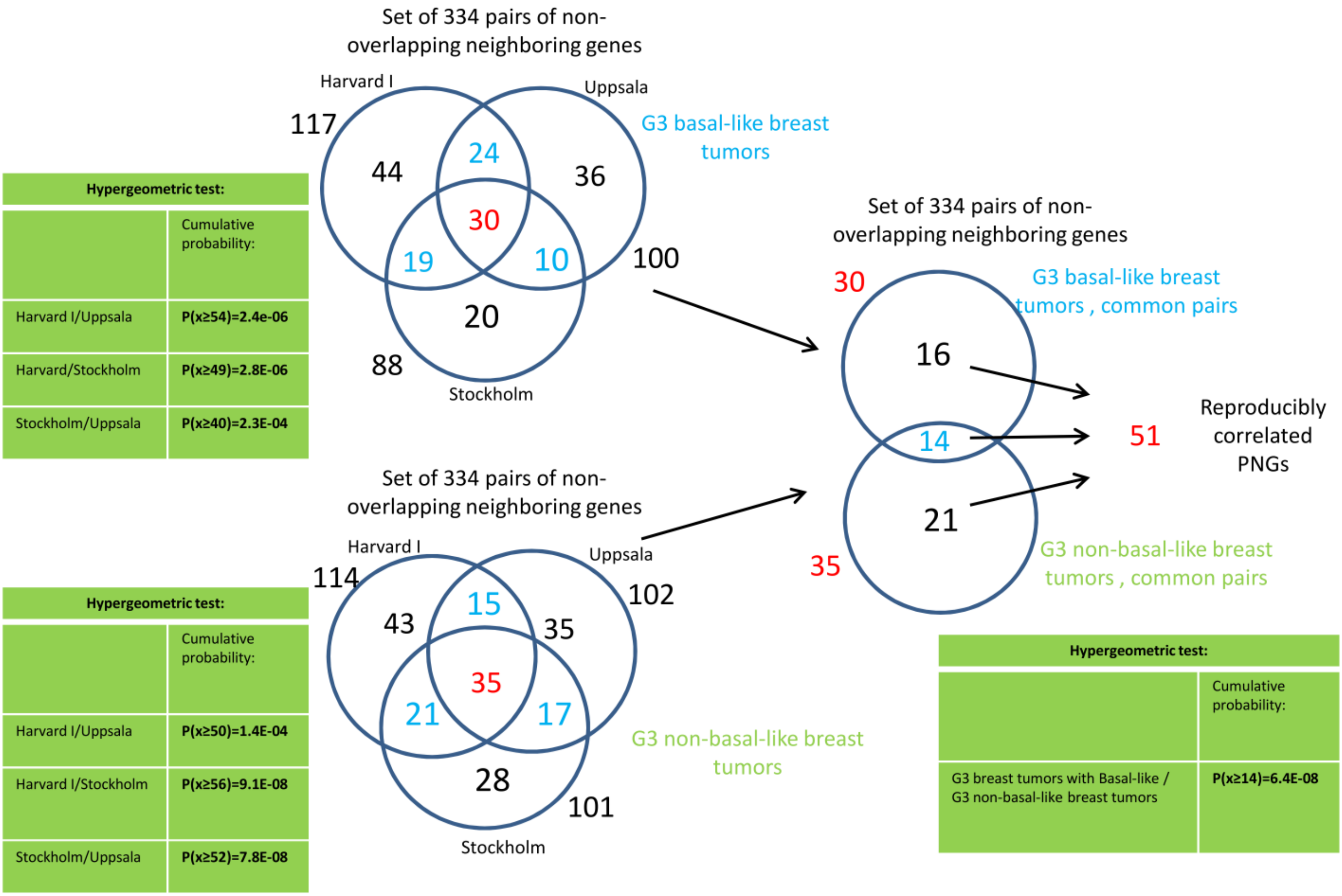

Hypergeometric test:	
	Cumulative probability:
Harvard I/Uppsala	**P(x≥54)=2.4e-06**
Harvard/Stockholm	**P(x≥49)=2.8E-06**
Stockholm/Uppsala	**P(x≥40)=2.3E-04**

Hypergeometric test:	
	Cumulative probability:
Harvard I/Uppsala	**P(x≥50)=1.4E-04**
Harvard I/Stockholm	**P(x≥56)=9.1E-08**
Stockholm/Uppsala	**P(x≥52)=7.8E-08**

Hypergeometric test:	
	Cumulative probability:
G3 breast tumors with Basal-like / G3 non-basal-like breast tumors	**P(x≥14)=6.4E-08**

Supplementary Figure S5: Workflow for identification of the prognostic sense-antisense gene signature (SAGS).

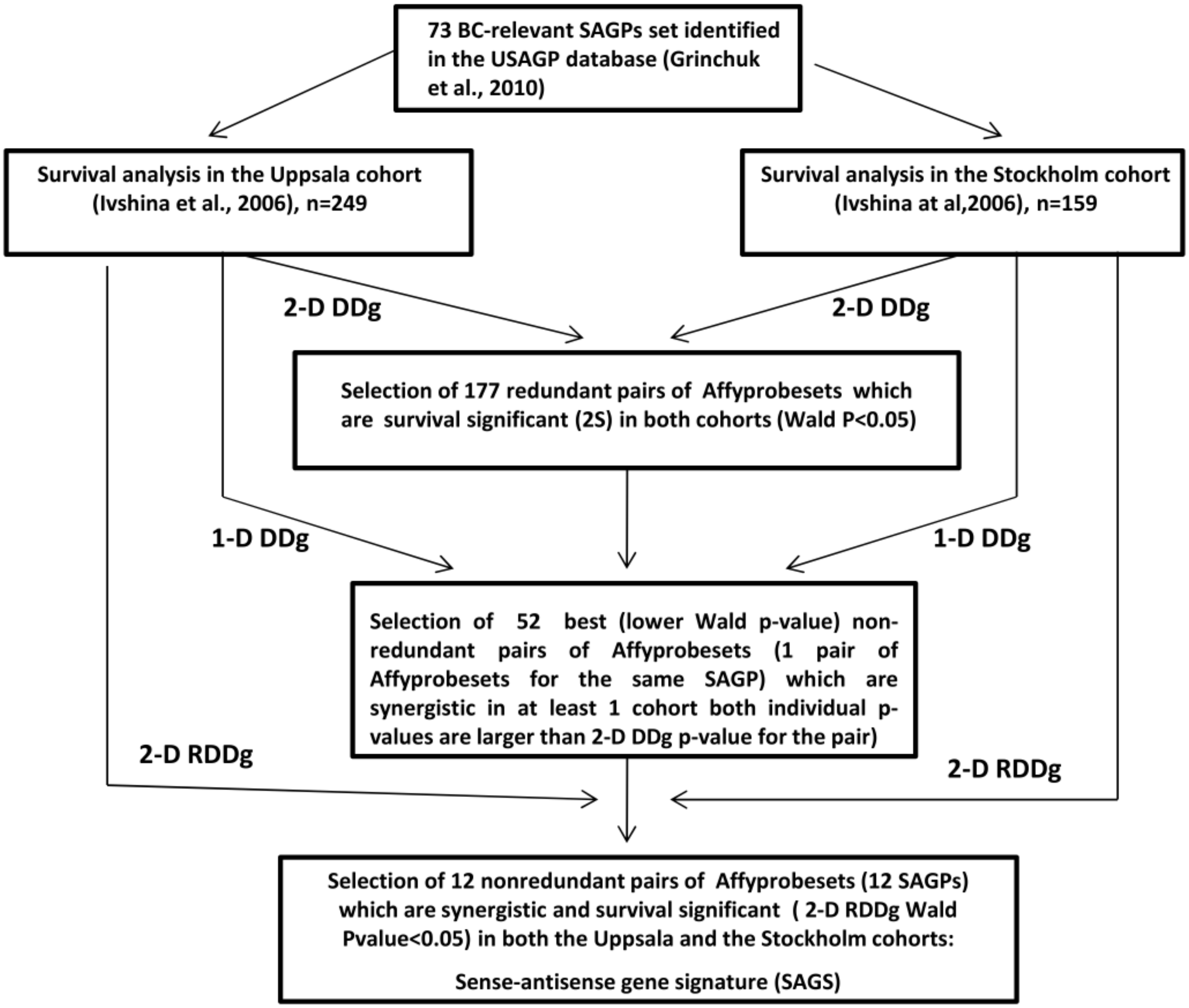

Supplementary Figure S6 2-D DDg: important components of the survival prediction method. (**A**) Grouping of gene pairs (genes 1 and 2 with respective cutoffs c^1 and c^2) and all possible the two-group designs (designs 1-5) used in the 2-D DDg. Red circles mark the sector of high risk of recurrence; black circles: the sector of low risk of recurrence. (**B**) All possible the two-group sub-designs.

A

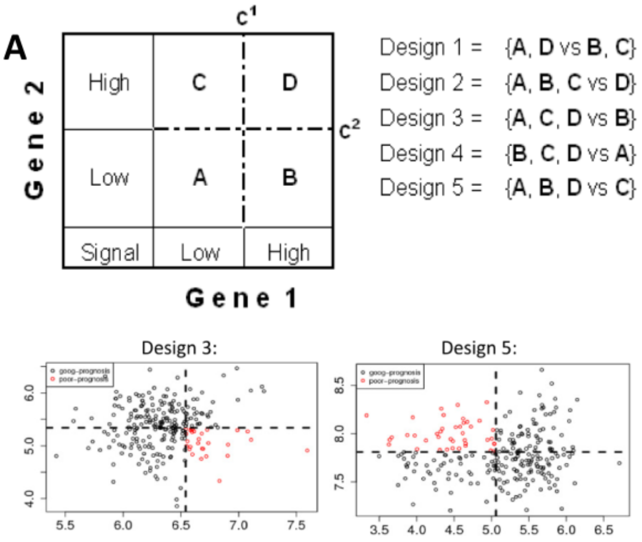

B

Subdesign1.1<br>Subdesign1.2	A, D (low risk patients) vs B, C (high risk patients)<br>A, D (high risk patients) vs B, C (low risk patients)
Subdesign2.1<br>Subdesign2.2	A, B, C (low risk patients) vs D (high risk patients)<br>A, B, C (high risk patients) vs D (low risk patients)
Subdesign3.1<br>Subdesign3.2	A, C, D (low risk patients) vs B (high risk patients)<br>A, C, D (high risk patients) vs B (low risk patients)
Subdesign4.1<br>Subdesign4.2	B, C, D (low risk patients) vs A (high risk patients)<br>B, C, D (high risk patients) vs A (low risk patients)
Subdesign5.1<br>Subdesign5.2	A, B, D (low risk patients) vs C (high risk patients)<br>A, B, D (high risk patients) vs C (low risk patients)

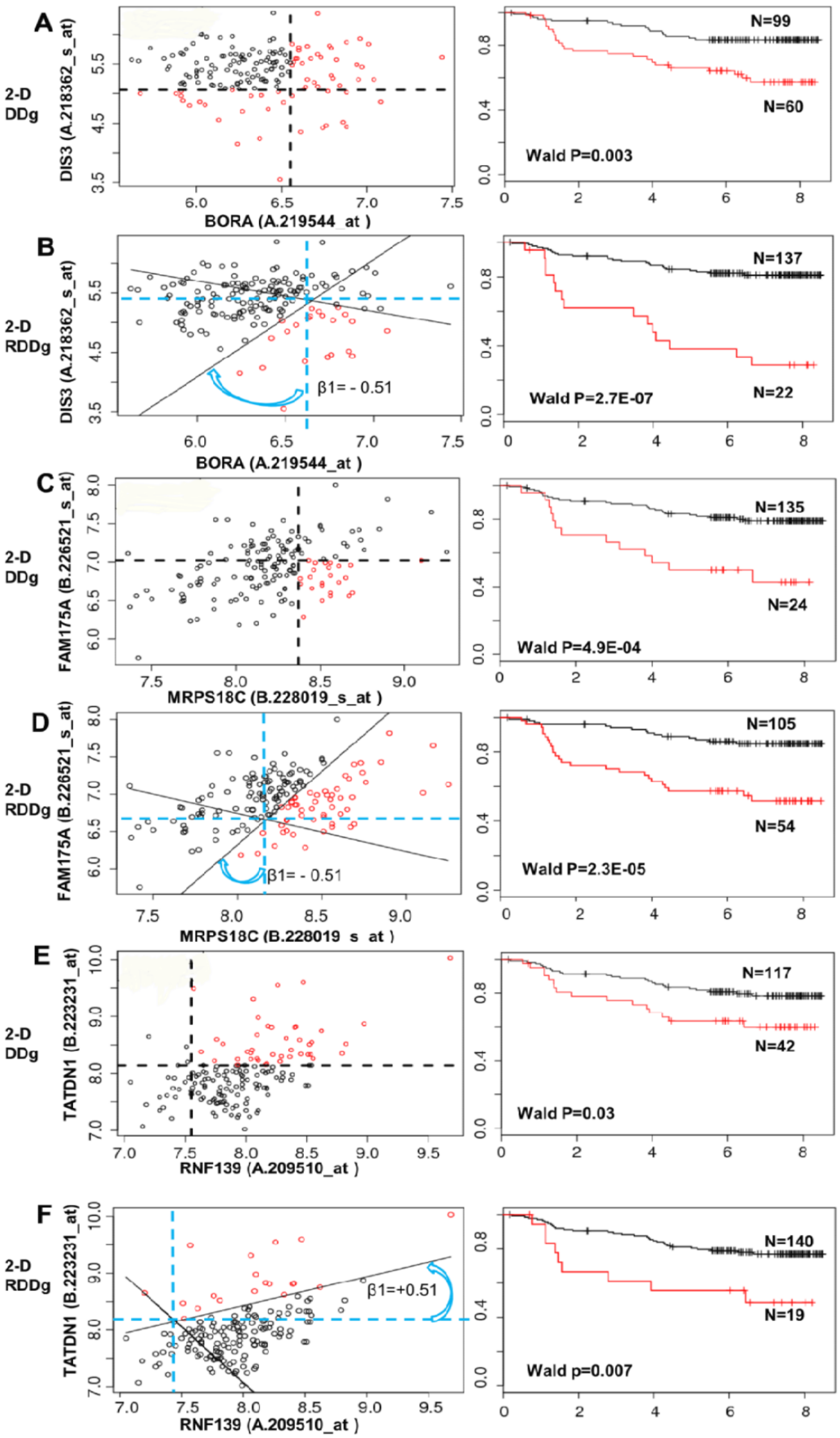

Supplementary Figure S7: Comparison of the results obtained by the 2-D RDDg and the 2-D DDg methods in 3 SAGPs belonging to the SAGS. In the same cohort (the Stockholm cohort, n=159) all the groupings were performed in the training mode. The SAGPs: (**A,B**) DIS3-BORA; (**C,D**) FAM175A-MRPS18C; (**E,F**) TATDN1-RNF139. **(A), (C)** and **(E)**: 2-D DDg; **(B)**, **(D)** and **(F)**: 2-D RDDg. Left panel: 2-dimentional plots of the patients partitions for each individual SAGP. Red circles: patients classified into the high-risk subgroup; black circles: patients classified into the low-risk subgroup. Blue dashed lines: original position of the orthogonal axes X and Y (and corresponding gene expression cutoffs) in 2-D RDDg before the rotation procedure (Figure 3, B). Blue arrow: direction of the consequent axes (X and Y) rotation to reach the most optimal partition at an angle β1. Right panel: the Kaplan-Meier survival plots for the individual SAGPs corresponding to the 2-dimentional plots on the left panel.

Supplementary Figure S8 ROC curves analysis of SAGS performance in 2 training (A) and (B) and 2 validation (C) and (D) BC cohorts**.**

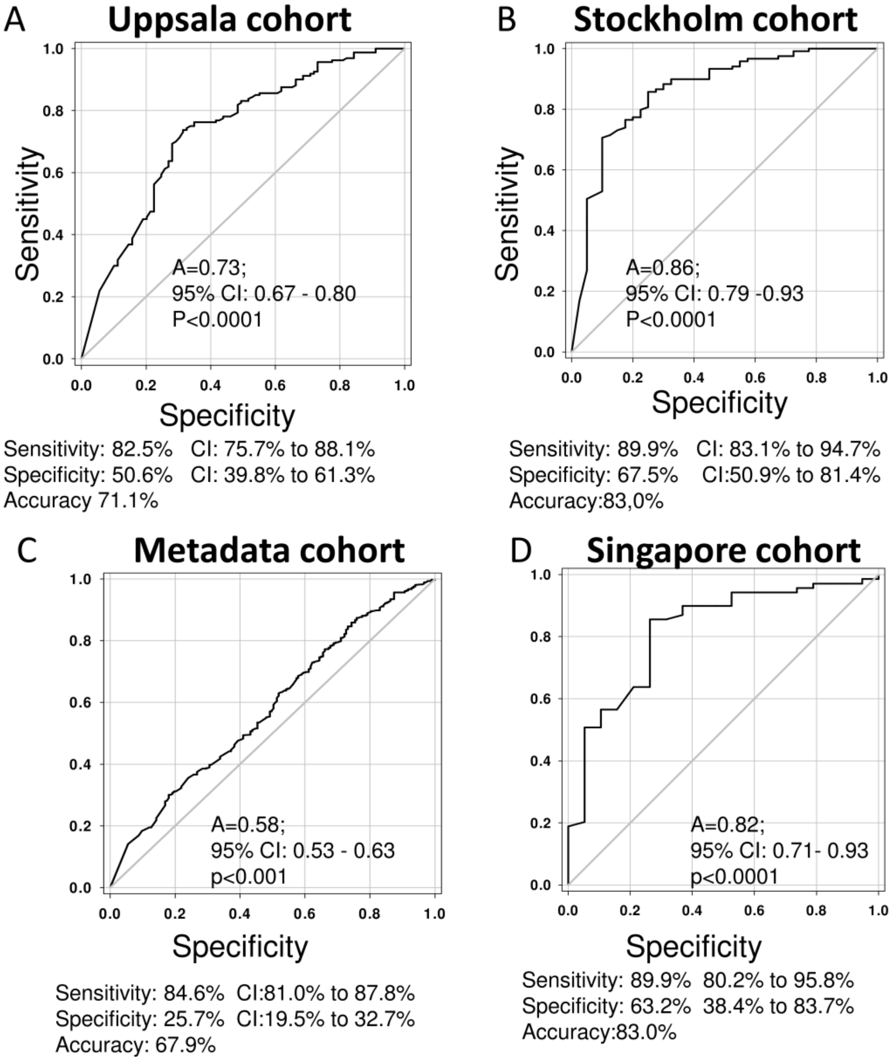

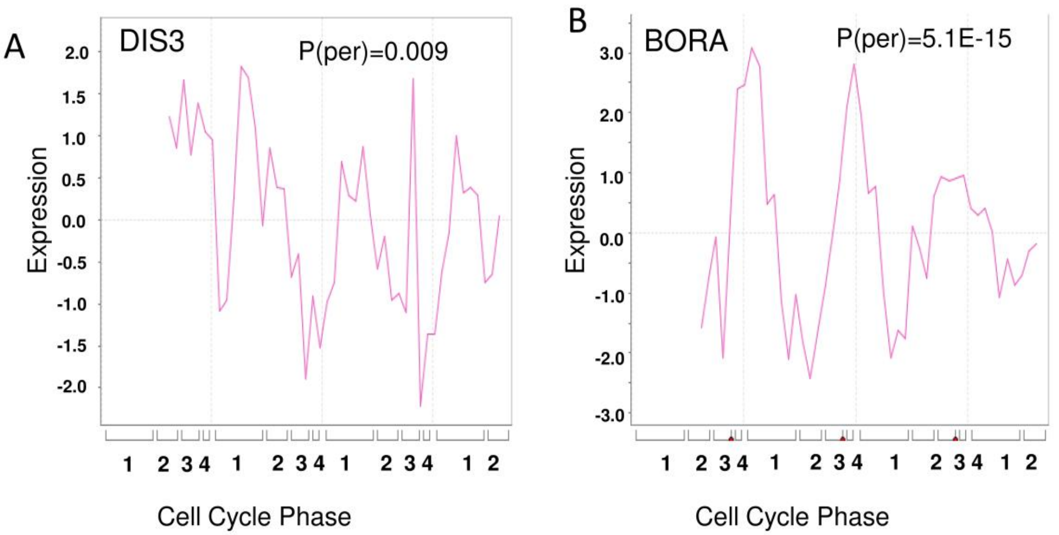

Supplementary Figure S9: *DIS3* and *BORA* expressions are coordinated in cell cycle phases.
(A and B) the same cDNA microarray gene expression time course experiment for the genes *DIS3* and *BORA* in HeLa cancer cells was assessed using the Cyclebase3.0 database. "1", "2", "3" and "4" denote G1, S, G2 and M phases of cell cycle, respectively. p-value for periodicity p(per) estimates the chance of observing a periodicity by random shuffling of the individual time-point values of the expression profile. (C and D) combined visualization of several available independent time course experiments for each gene.

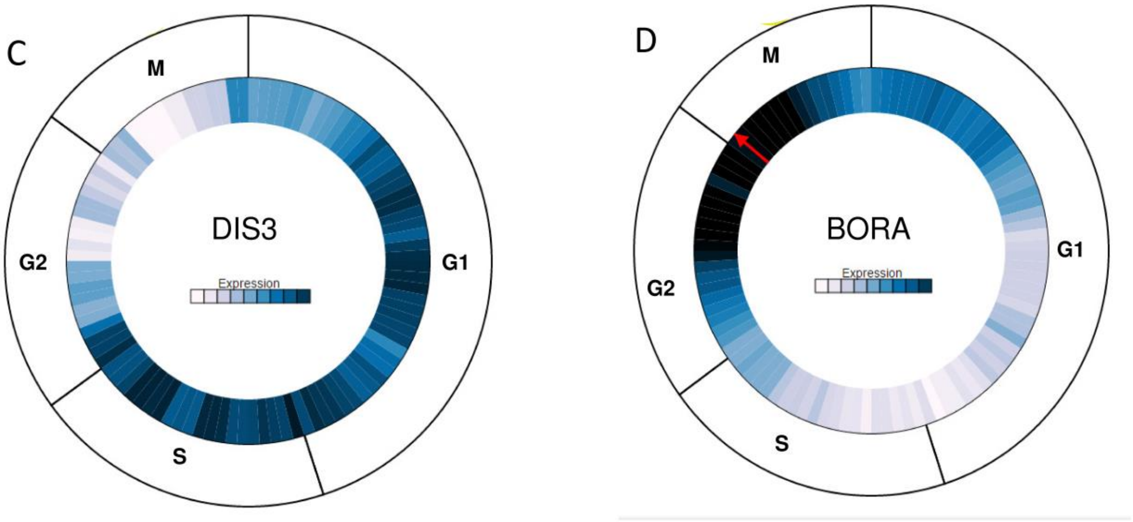

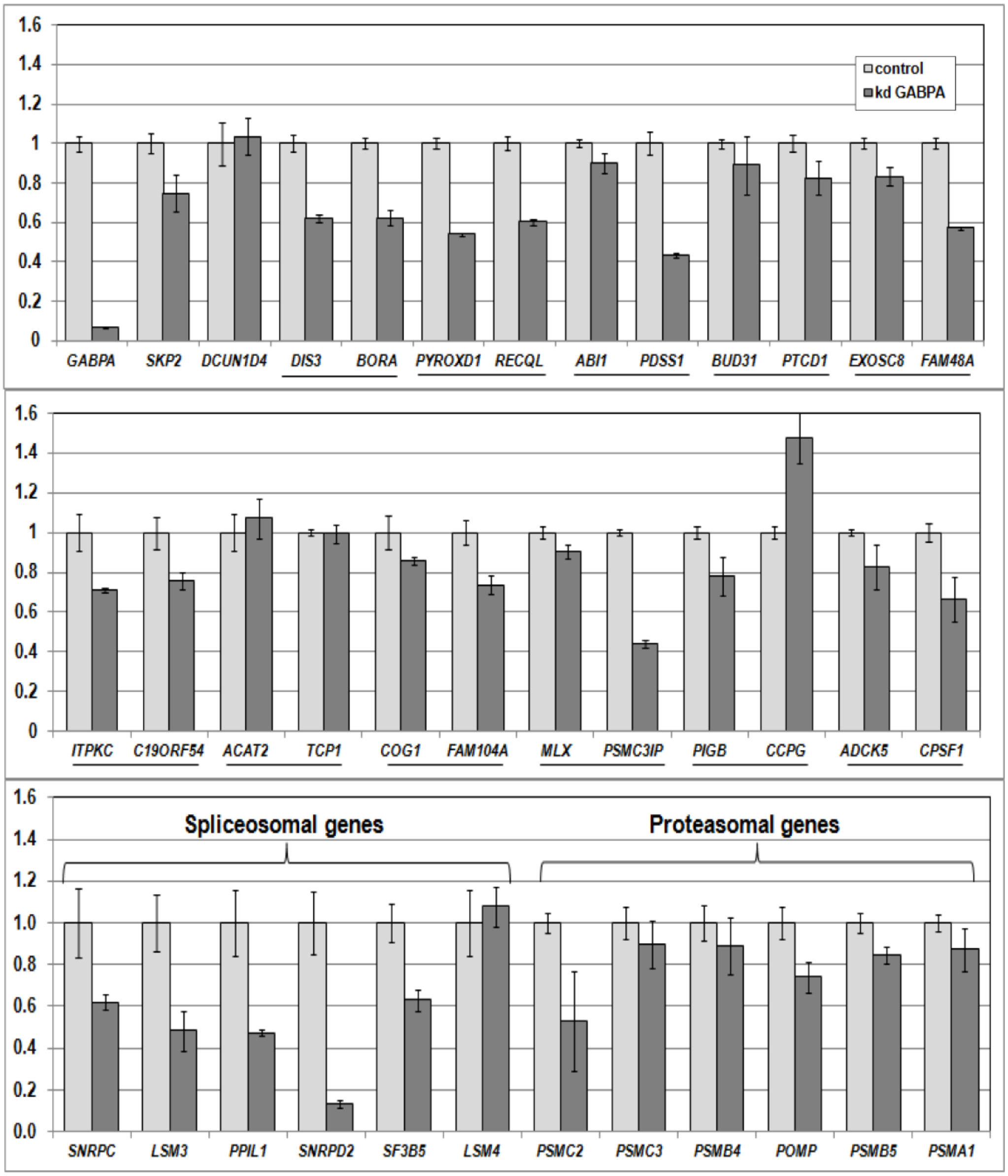

Supplementary Figure S10: Effects of siRNA *GABPA* knockdown in 35 genes from the *GABPA* gene network (MCF-7 cells). First and second rows: *SKP2* is the known direct effector of *GABPA*, promoter of which overlaps with *GABPA* CBR (see Discussion); *DCUN1D4* gene (negative control) is highly expressed in MCF-7 cells but its promoter does not overlap with an *GABPA* CBR. Eleven randomly selected convergent BCR-ccSAGPs are shown in which both gene proximal promoters overlap with *GABPA* CBRs (underlined). Third row: *GABPA* knockdown in 12 randomly selected spliceosomal and proteasomal genes from the *GABPA* gene network. Y-axis: relative gene expression between intact MCF-7 cells (arbitrarily set at 1) and cells with *GABPA* knockdown using *GABPA* siRNA. Gene expression change between 2 experiments was calculated using ΔΔCt method (see Materials and Methods). Light grey bars: intact MCF-7 cells; dark grey bars: MCF-7 cells with GABPA knockdown. All measurements were performed in triplicates.
